# Supplementary material for: CTCF controls three-dimensional enhancer network underlying the inflammatory response of bone marrow-derived dendritic cells
Source: Nat Commun. 2023 Mar 8;14:1277. doi: 10.1038/s41467-023-36948-5 (PMC9992691; doi:10.1038/s41467-023-36948-5)
Supplement: Supplementary file 1 — Supplementary Information [file 41467_2023_36948_MOESM1_ESM.pdf]

Supplementary information

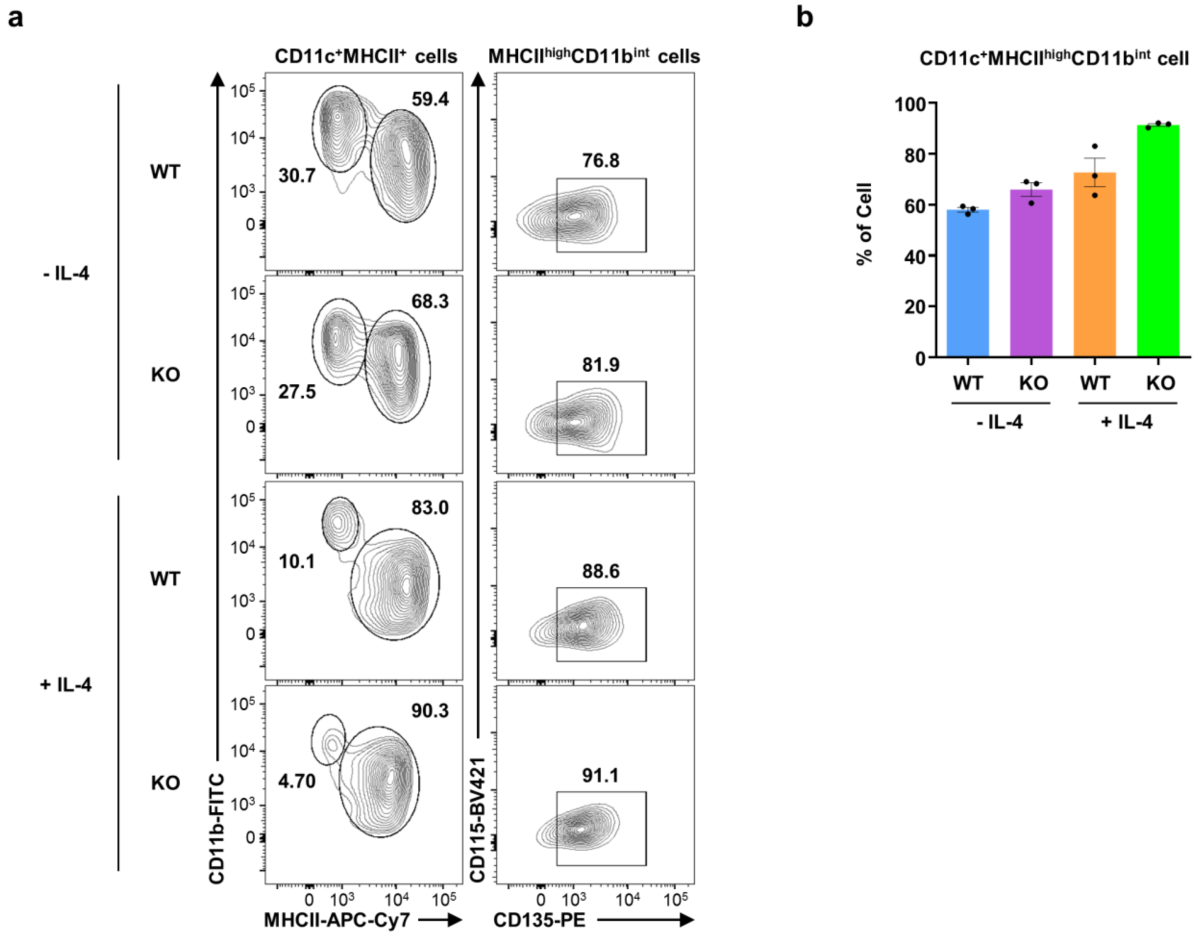

Supplementary Figure 1. (related to Figure 1) Phenotype of cells developing in GM-CSF BM cultures. a Phenotype of representative GM-CSF BM cultures with or without IL-4 at day 6. CD11c^{+}MHCII^{+} cells can be sub-divided on the basis of CD11b and MHCII expression (left), which can further be sub-divided on the basis of CD135 and CD115 expression (right). Boxes depict gates and numbers correspond to percentage of cells in each gate. Data are representative of three independent experiments. **b** Summarized bar graph for percentage of CD11c^{+}MHCII^{high}CD11b^{int} BMDCs. Error bars represent mean ± standard error of the mean (s.e.m). *n*=3 biologically independent samples. Source data are provided as a Source Data file.

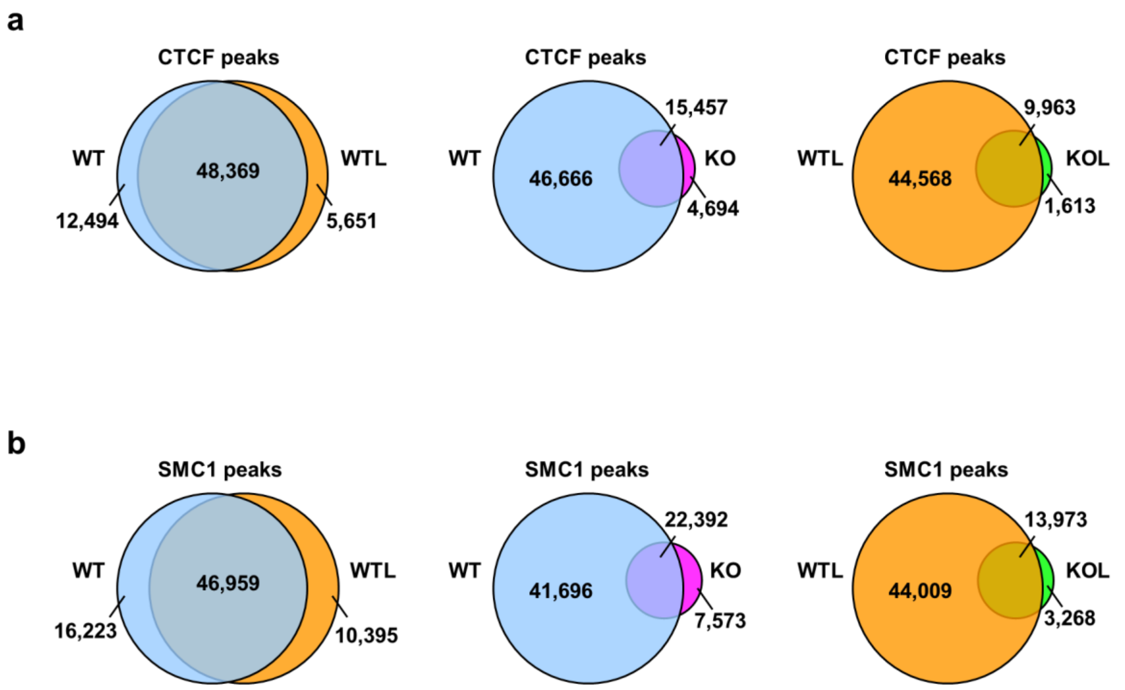

Supplementary Figure 2. (related to Figure 1) Disrupted occupancies of CTCF and SMC1 due to CTCF depletion. a,b Overlap of CTCF (**a**) and SMC1 (**b**). ChIP-Seq peaks between WT and WTL (left), between WT and KO (middle), or between WTL and KOL (right). WT: untreated wild-type BMDC, WTL: wild-type BMDC treated with LPS for 3 hours, KO: untreated CTCF knock-out BMDC, KOL: CTCF knock-out BMDC treated with LPS for 3 hours.

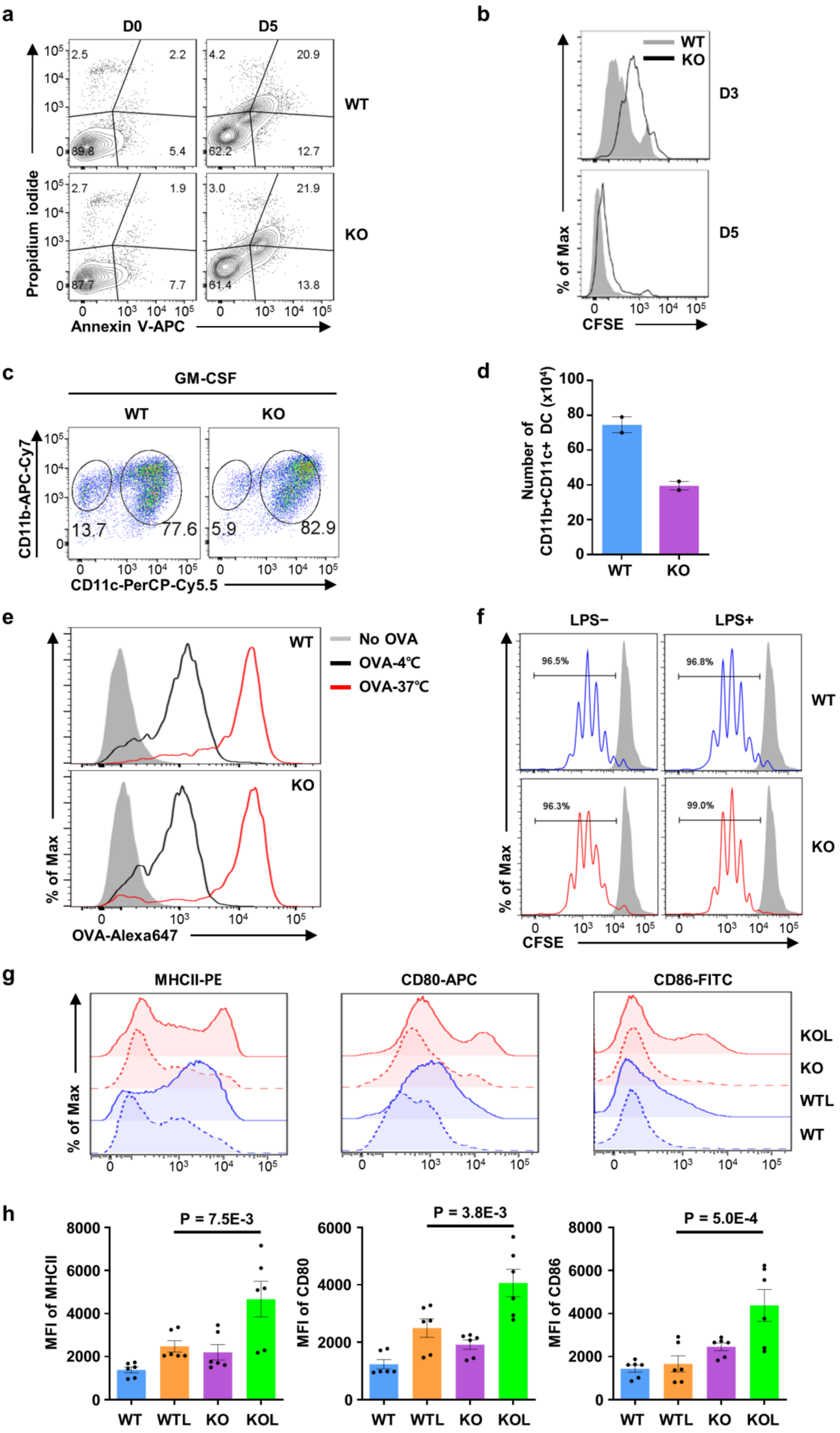

a
D0
D5
WT
KO
Propidium iodide
Annexin V-APC
2.5
2.2
89.8
5.4
4.2
20.9
62.2
12.7
2.7
1.9
87.7
7.7
3.0
21.9
61.4
13.8
b
WT
KO
D3
D5
% of Max
CFSE
c
GM-CSF
WT
KO
CD11b-APC-Cy7
CD11c-PerCP-Cy5.5
13.7
77.6
5.9
82.9
d
Number of CD11b+CD11c+ DC (x10⁴)
WT
KO
e
No OVA
OVA-4℃
OVA-37℃
WT
KO
% of Max
OVA-Alexa647
f
LPS−
LPS+
96.5%
96.8%
96.3%
99.0%
WT
KO
% of Max
CFSE
g
MHCII-PE
CD80-APC
CD86-FITC
KOL
KO
WTL
WT
% of Max
h
P = 7.5E-3
P = 3.8E-3
P = 5.0E-4
MFI of MHCII
MFI of CD80
MFI of CD86
WT
WTL
KO
KOL

Supplementary Figure 3. (related to Figure 1) CTCF is dispensable for overall differentiation and survival of BMDCs. a Representative flow cytometric plots of the frequency of live cells during GM-CSF-supplemented BM culture. **b** Representative flow cytometric plots of the CFSE-diluted proliferating population during GM-CSF-supplemented BM culture at the indicated time points. **c, d** Representative flow cytometric plots (**c**) and summarized bar graph for the number (**d**) of GM-CSF-induced $CD11c^{+}CD11b^{+}$ DCs from WT and KO BM. Error bars represent mean ± standard error of the mean (s.e.m). *n*=2 biologically independent samples. **e** Representative flow cytometric plots of the OVA-Alexa647 antigen uptake by WT and KO BMDCs. **f** Representative flow cytometric plots of CFSE-diluted antigen-specific $CD4^{+}$ OT-II T cell proliferation in response to OT-II peptide-pulsed WT and KO BMDCs in vitro. **g, h** Representative flow cytometric plots (**g**) of the expression level of surface MHC II, CD80, and CD86 and (**h**) summarized bar graph for mean fluorescence intensity (MFI) from WT and KO DCs. Error bars represent mean ± standard error of the mean (s.e.m). Significance was calculated using a two-way ANOVA with multiple comparisons of Bonferroni post-test using *n*=3 independent samples. Source data are provided as a Source Data file.

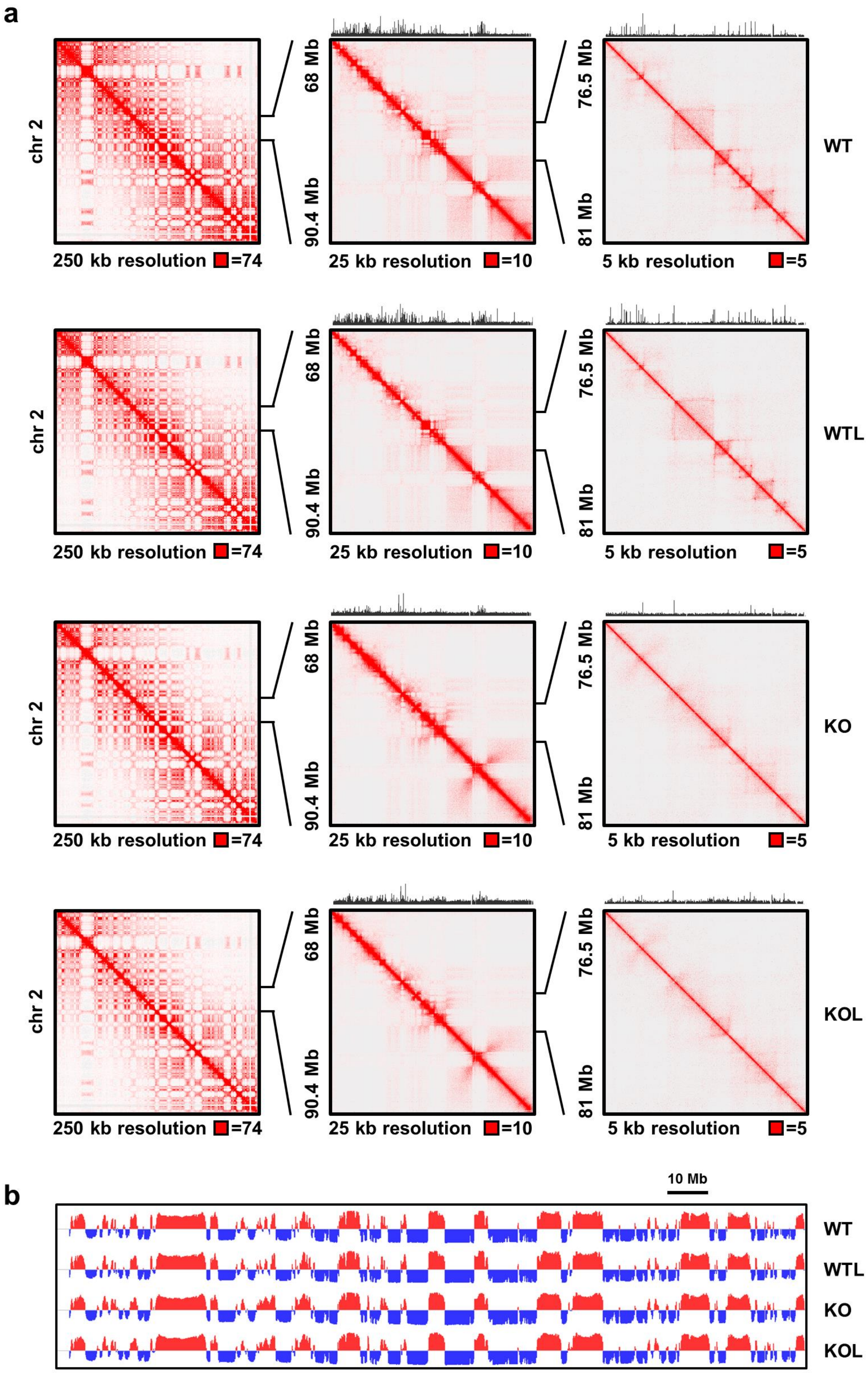
a
chr 2
68 Mb
90.4 Mb
76.5 Mb
81 Mb
250 kb resolution =74
25 kb resolution =10
5 kb resolution =5
WT
WTL
KO
KOL
b
10 Mb
WT
WTL
KO
KOL

Supplementary Figure 4. (related to Figure 2) Compartments are largely unaffected by either LPS stimulation or CTCF depletion. a Hi-C contact maps generated by Juicebox at 250 kb, 25 kb, and 5 kb resolutions. CTCF ChIP-seq signal tracks were aligned on top of the contact maps at 25 kb and 5 kb resolutions. Numbers below the interaction maps correspond to the maximum signal in the matrix. **b** Distributions of cis Eigenvector 1 values across the entirety of chromosome 16.

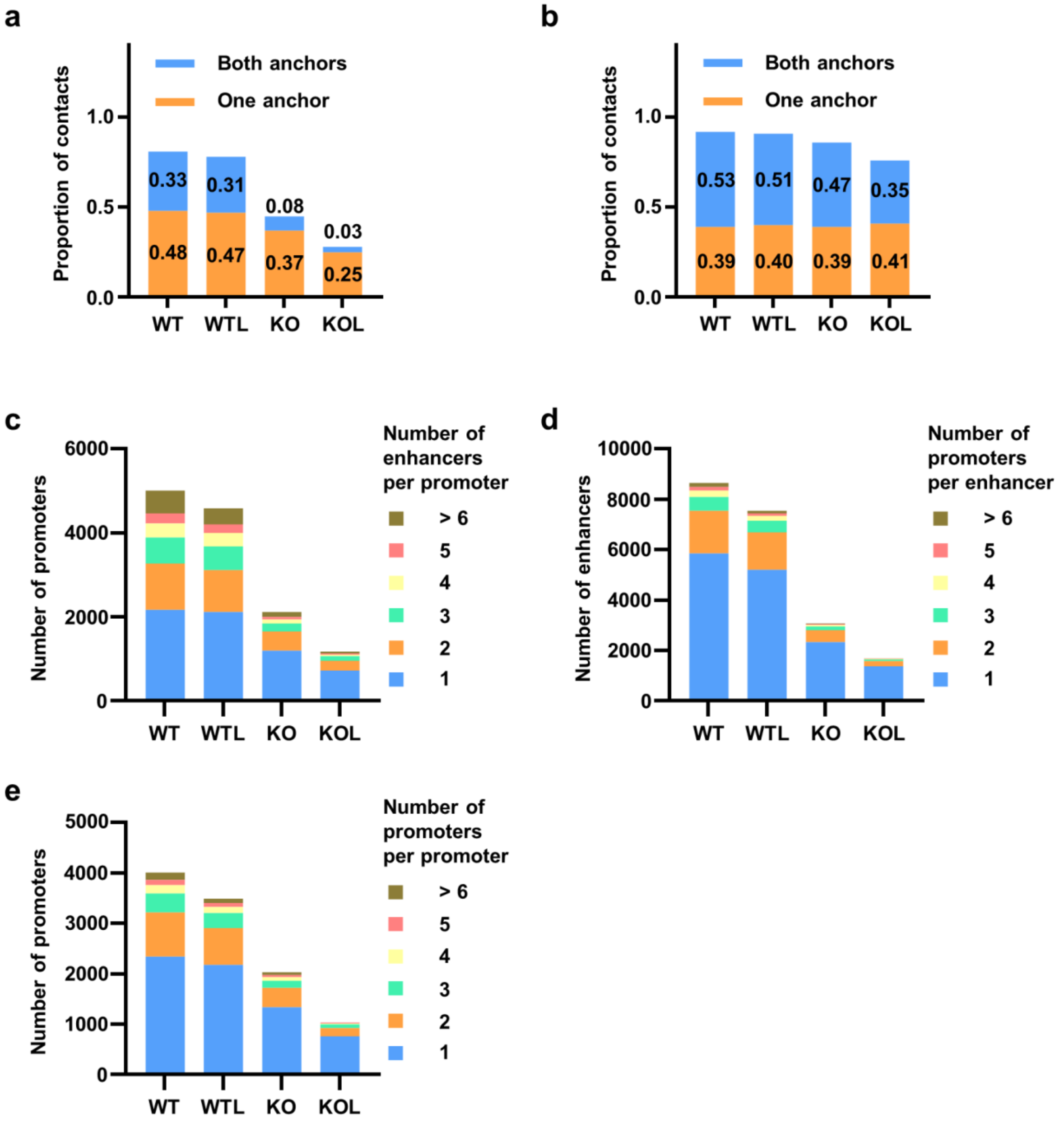

Supplementary Figure 5. (related to Figure 2) Enhancer-promoter interactions called from H3K27ac HiChIP. a, b Percentage of overlap of CTCF (**a**) and SMC1 (**b**) ChIP-seq peaks with either one or both H3K27ac HiChIP loop anchors. **c** The number of enhancer-interacting promoters. **d** The number of promoter-interacting enhancers. **e** The number of promoter-interacting promoters.

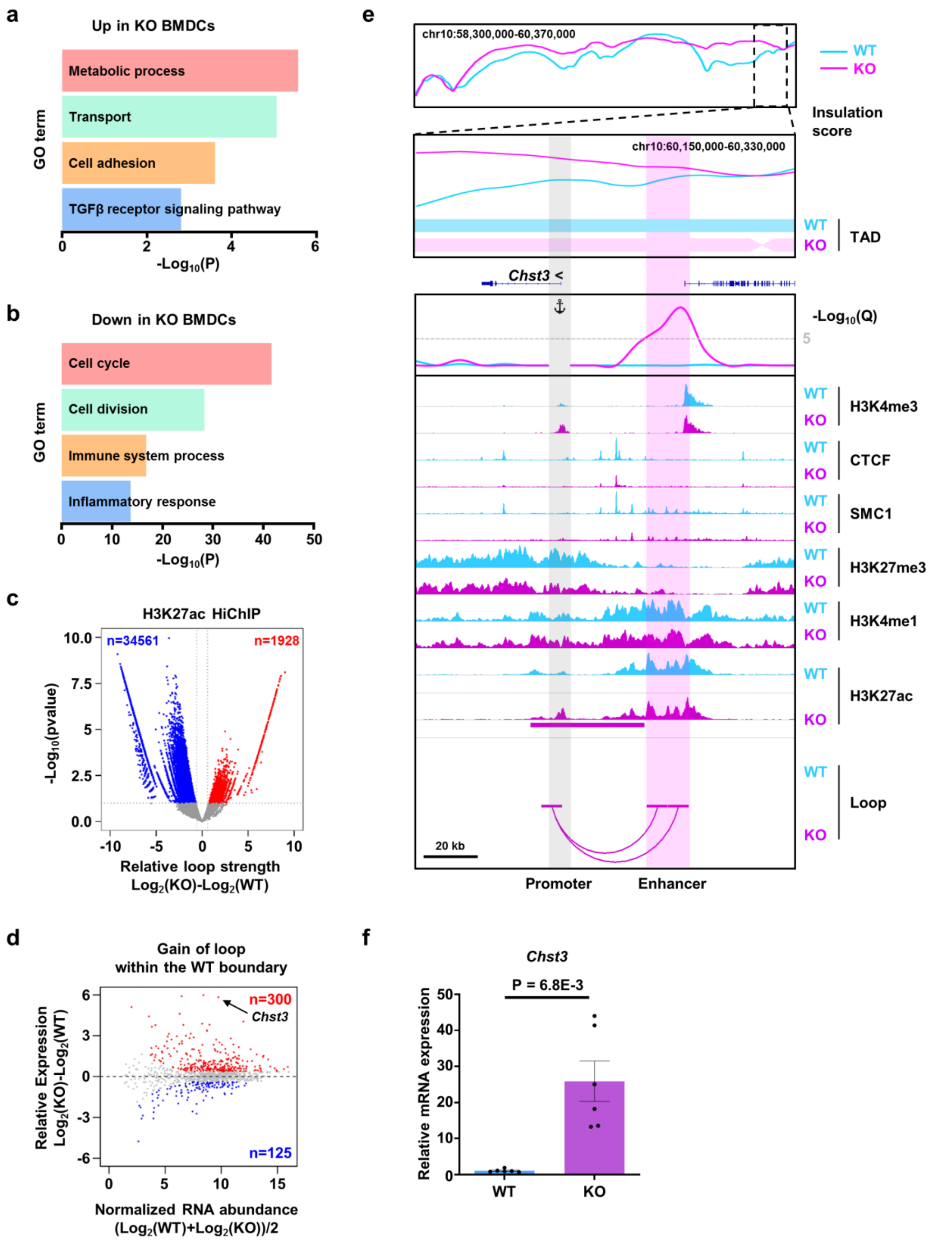

Supplementary Figure 6. (related to Figure 4) Gain of loops to already-active distal enhancers due to CTCF depletion leads to dysregulated gene expression. a, b Enrichment of pathway terms on the genes whose RNA expression was upregulated (a) or

downregulated (b) in KO BMDCs, are shown as bar plots of –Log10 *P* value. Significance in (a, b) was calculated by one-sided Fisher's Exact test. **c** Volcano plot showing differential H3K27ac HiChIP loops between WT and KO BMDCs. The number of loops exhibiting >1.5-fold in WT (blue) or KO (red) BMDCs with a *p*-value <0.1 has been indicated. **d** RNA-seq MA plot for the genes associated with "gain of loop within the WT boundary". Mean abundance is plotted on the *x* axis and enrichment is plotted on the *y* axis. The number of genes that exhibited increases of greater than 1.3-fold in WT (blue) or KO (red) BMDCs with a false discovery rate of less than 0.05 have been indicated. **e** Snapshots displaying insulation score curves with TAD boundary, virtual 4C plots, ChIP-seq signal tracks, and significant loops (from top to bottom) at the *Chst3* locus. Sky blue and purple represent the wild-type BMDCs (WT) and CTCF knockout BMDCs (KO), respectively. Virtual 4C plots (V4C) shows normalized H3K27ac HiChIP loop strength (represented as -Log10(*Q*)) with the promoter of *Chst3* gene as the viewpoint. IGV browser shows ChIP-seq signal tracks for H3K4me3, CTCF, SMC1, H3K27me3, H3K4me1, and H3K27ac. Arcs display significant interactions with –Log10(*Q*) ≥ 5. Only loops interacting with the viewpoint have been displayed. Gray vertical bars highlight the location of promoters used as viewpoints. Purple vertical bars highlight the location of distal enhancers interacting with viewpoint *Chst3* (d) promoter in KO. **f** Relative mRNA expression level for *Chst3* gene. Error bars represent mean ± standard error of the mean (s.e.m). Significance was calculated using an unpaired two-tailed *t*-test using *n*=3 independent samples. Source data are provided as a Source Data file.

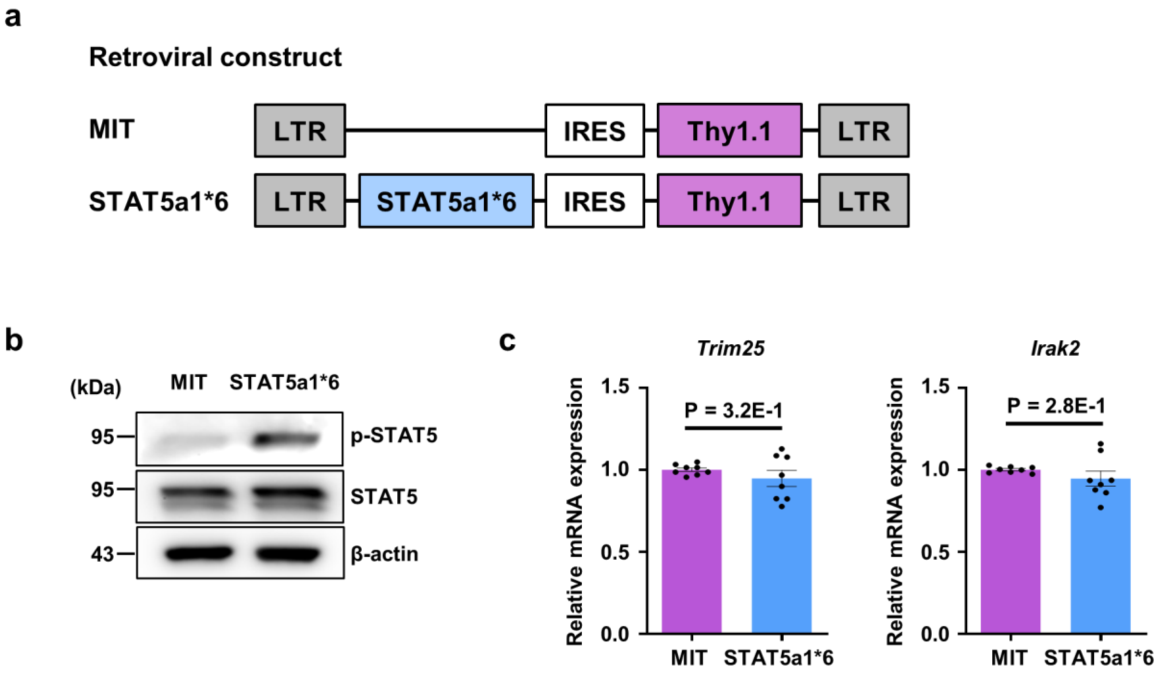

Supplementary Figure 7. (related to Figure 5) Restoration of STAT5 activity in CTCF-deficient BMDCs. a Schematic showing MSCV retroviral vector expressing constitutively-active STAT5 (STAT5a1*6) or empty vector (MIT). **b** KO BMDCs were infected with retrovirus and western blotting was performed with indicated antibodies. The data were representative of three independent experiments with similar results. **c** Relative mRNA expression levels for *Trim25* and *Irak2* gene. Error bars represent mean ± standard error of the mean (s.e.m). Significance was calculated using an unpaired two-tailed *t*-test using *n*=3 independent samples. Source data are provided as a Source Data file.

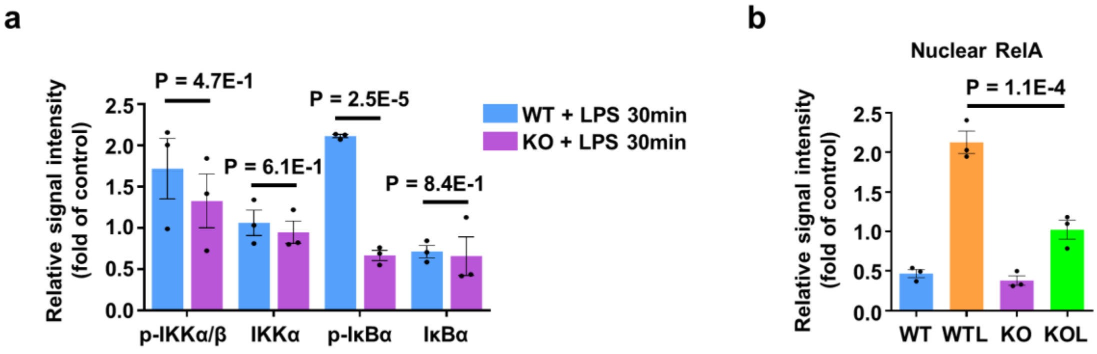

Supplementary Figure 8. (related to Figure 6) Attenuation of LPS-induced NF-κB activation in CTCF-deficient BMDCs. a,b Relative signal intensities of proteins in Fig. 6a (**a**) and Fig. 6d (**b**) were measured using ImageJ software. Error bars represent mean ± standard error of the mean (s.e.m). Significance in (**a**) was calculated using an unpaired two-tailed *t*-test using *n*=3 independent samples. Significance in (**b**) was calculated using a two-way ANOVA with multiple comparisons of Bonferroni post-test using *n*=3 independent samples. Source data are provided as a Source Data file.

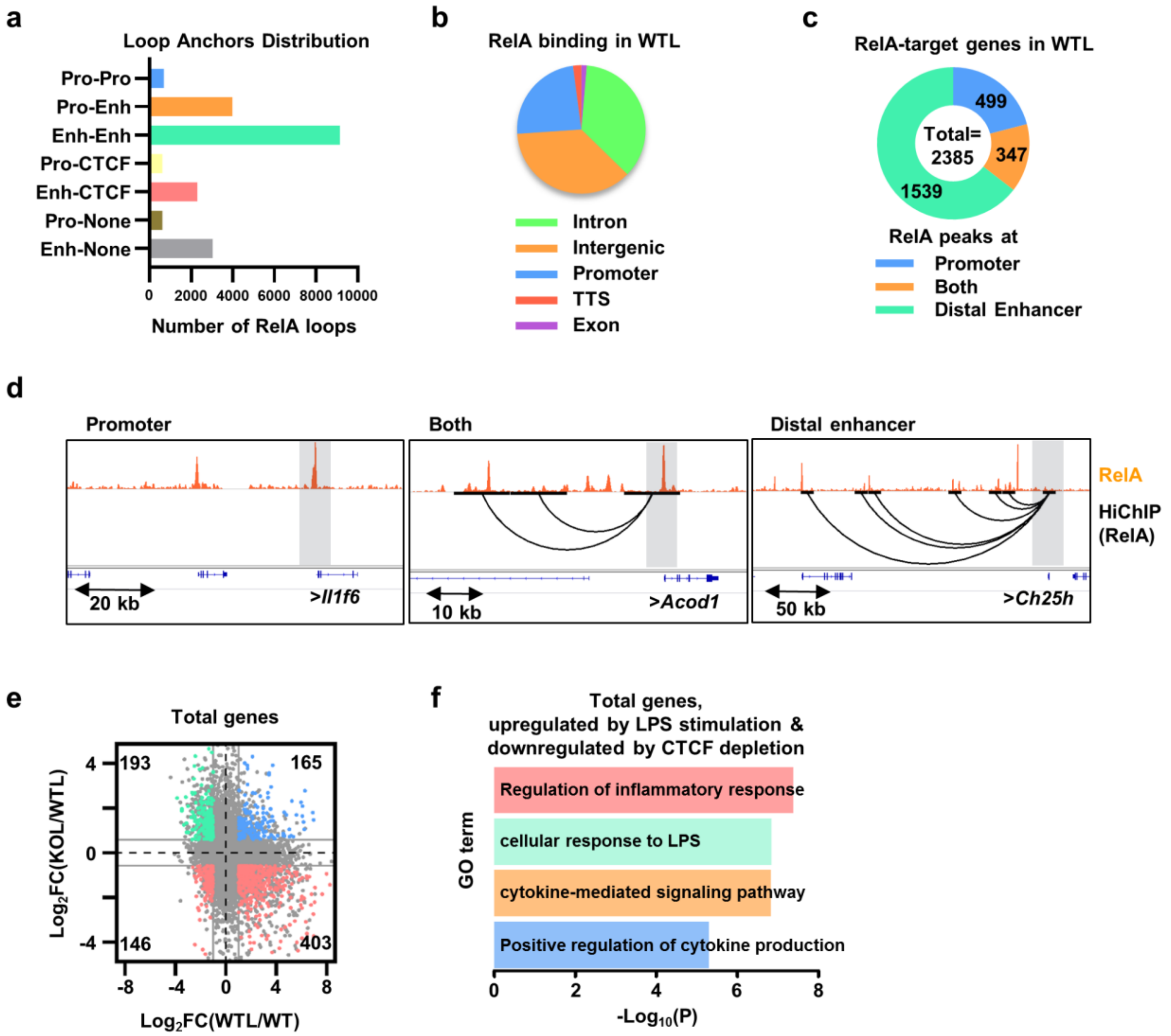

Supplementary Figure 9. (related to Figure 7) Mapping NF-κB-bound chromatin loops in LPS-stimulated WT BMDCs. a Distribution of regulatory elements at the anchors of high-confidence RelA HiChIP loops. **b** Pie charts presenting the distribution of RelA ChIP-seq peaks called from WTL among different gene features. **c** A pie chart depicting the number of RelA direct target genes in LPS-stimulated wild-type BMDCs (WTL), classified by the overlap feature of RelA peaks with promoters, distal enhancers, or both. **d** Snapshots shows RelA ChIP-seq signal tracks and significant RelA HiChIP loops at the typical RelA direct target genes classified by the overlap feature of RelA peaks with promoters, distal enhancers, and both. Arcs shows significant interactions with –Log10(Q) ≥ 2. Gray vertical bars highlight the location of TSSs used as viewpoints. Only loops interacting with the viewpoint have been

displayed. **e** Scatter plot showing Log2-fold changes in RNA expression levels of total genes. The number of genes exhibiting >2-fold changes in WTL versus WT and 1.5-fold changes in KOL versus WTL with a false discovery rate <0.05 is indicated at each quartile. **f** Bar plot of –Log10 *P* value showing enrichment of gene ontology terms on the genes whose expressions were upregulated by LPS stimulation and downregulated by CTCF depletion. Significance was calculated by one-sided Fisher's Exact test. Source data are provided as a Source Data file.

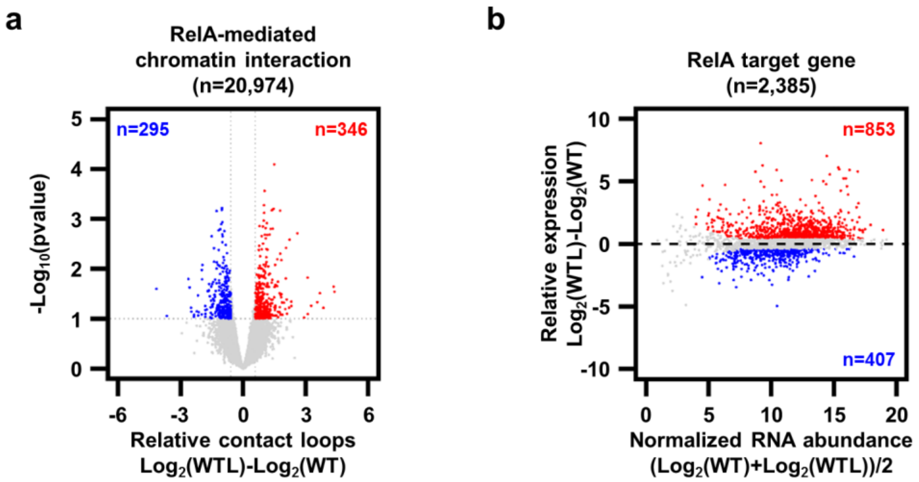

Supplementary Figure 10. (related to Figure 8) NF-κB signaling control enhancer activation and enhancer-promoter interaction for the optimal expression of its target genes. a Volcano plot of H3K27ac HiChIP of untreated WT BMDCs (WT) versus LPS-stimulated WT BMDCs (WTL) for showing significant changes in RelA-mediated chromatin interactions in response to LPS stimulation. The number of loops exhibiting >1.5-fold increases in WT (blue) or WTL (red) BMDCs with a *p*-value <0.1 has been indicated. **b** RNA-seq MA plot of untreated WT BMDCs (WT) versus LPS-stimulated WT BMDCs (WTL) for showing significant changes in the transcript abundance of RelA target genes in response to LPS stimulation. The number of genes exhibiting >2-fold increases in WT (blue) or WTL (red) BMDCs with a false discovery rate <0.05 has been indicated. Source data are provided as a Source Data file.

Supplementary Table 1. List of primers used in the study

Primers used for qRT-PCR		
**Name**	**Description**	**Sequence (5'-3')**
**Rpl7_F**	qRT-PCR	ATGTGCCCGCAGAACCAA
**Rpl7_R**	qRT-PCR	GACGAAGGAGCTGCAGAACCT
**Ctcf_F**	qRT-PCR	AGCGCTATCATGATCCCAAC
**Ctcf_R**	qRT-PCR	CGGCTCAGCATTTTCTTCAC
**Aldh1a2_F**	qRT-PCR	ATGGGTGAGTTTGGCTTACG
**Aldh1a2_R**	qRT-PCR	GGTTCATTGGAAGGCAGAAA
**Il6_F**	qRT-PCR	CCGGAGAGGAGACTTCACAG
**Il6_R**	qRT-PCR	TTGCCATTGCACAACTCTTT
**Il12a_F**	qRT-PCR	GGCCATCAACGCAGCACTTC
**Il12a_R**	qRT-PCR	CACAGGAGGTTTCTGGCGCA
**Il12b_F**	qRT-PCR	CCTGAAGTGTGAAGCACCAA
**Il12b_R**	qRT-PCR	GTCAGGGGAACTGCTACTGC
**Chst3_F**	qRT-PCR	CTCACAACTTTCCCACAGCA
**Chst3_R**	qRT-PCR	AGCTCAGGAGCATAGCTTGG
**Trim25_F**	qRT-PCR	CGGAAAATTCGACACCATCT
**Trim25_R**	qRT-PCR	CCTTGCAATTTTGCAGCTTT
**Irak2_F**	qRT-PCR	CCTACATGGCAAATGGCTCT
**Irak2_R**	qRT-PCR	TGTGGATGATGTCCAGGCTA

Supplementary Table 2. Summary of Hi-C data

	WT rep1	WT rep2	Merge WT	WTL rep1	WTL rep2	Merge WTL
**Total_pairs_processed**	390,283,588	406,459,496	796,743,084	380,957,831	376,456,002	757,413,833
**Unmapped_pairs**	11,468,239	10,439,894	21,908,133	17,123,457	8,866,667	25,990,124
**Low_qual_pairs**	90,161,418	96,398,078	186,559,496	80,643,306	83,276,537	163,919,843
**Unique_paired_alignments**	263,990,885	274,469,261	538,460,146	262,053,667	264,830,214	526,883,881
**Multiple_pairs_alignments**	0	0	0	0	0	0
**Pairs_with_singleton**	24,663,046	25,152,263	49,815,309	21,137,401	19,482,584	40,619,985
**Low_qual_singleton**	0	0	0	0	0	0
**Unique_singleton_alignments**	0	0	0	0	0	0
**Multiple_singleton_alignments**	0	0	0	0	0	0
**Reported_pairs**	263,990,885	274,469,261	538,460,146	262,053,667	264,830,214	526,883,881
**valid_interaction**	216,872,060	239,680,731	456,552,791	182,712,507	210,700,660	393,413,167
**valid_interaction_rmdup**	189,072,423	209,163,589	398,222,331	148,495,074	178,711,528	327,188,519
**trans_interaction**	44,062,168	47,101,172	91,160,541	29,208,608	33,498,463	62,704,056
**cis_interaction**	145,010,255	162,062,417	307,061,790	119,286,466	145,213,065	264,484,463
**cis_shortRange**	26,480,537	29,285,964	55,760,814	25,672,239	30,830,789	56,494,032
**cis_longRange**	118,529,718	132,776,453	251,300,976	93,614,227	114,382,276	207,990,431
	**KO rep1**	**KO rep2**	**Merge KO**	**KOL rep1**	**KOL rep2**	**Merge KOL**
**Total_pairs_processed**	412,220,990	376,032,438	788,253,428	398,575,970	409,017,683	807,593,653
**Unmapped_pairs**	16,419,477	11,060,748	27,480,225	8,845,555	12,366,980	21,212,535
**Low_qual_pairs**	90,366,695	84,847,514	175,214,209	93,165,865	83,975,603	177,141,468
**Unique_paired_alignments**	268,555,085	251,172,513	519,727,598	270,568,985	278,847,562	549,416,547
**Multiple_pairs_alignments**	0	0	0	0	0	0
**Pairs_with_singleton**	36,879,733	28,951,663	65,831,396	25,995,565	33,827,538	59,823,103
**Low_qual_singleton**	0	0	0	0	0	0
**Unique_singleton_alignments**	0	0	0	0	0	0
**Multiple_singleton_alignments**	0	0	0	0	0	0
**Reported_pairs**	268,555,085	251,172,513	519,727,598	270,568,985	278,847,562	549,416,547
**valid_interaction**	205,863,143	198,395,863	404,259,006	228,128,770	199,474,784	427,603,554
**valid_interaction_rmdup**	173,418,254	172,990,445	346,393,407	198,476,868	155,630,451	354,088,027
**trans_interaction**	41,039,979	41,550,778	82,587,405	47,211,086	42,129,149	89,336,153
**cis_interaction**	132,378,275	131,439,667	263,806,002	151,265,782	113,501,302	264,751,874
**cis_shortRange**	29,705,804	27,752,510	57,450,727	32,210,615	25,315,748	57,518,005
**cis_longRange**	102,672,471	103,687,157	206,355,275	119,055,167	88,185,554	207,233,869

Supplementary Table 3. Summary of H3K27ac HiChIP

	WT rep1	WT rep2	Merge WT	WTL rep1	WTL rep2	Merge WTL
**Total_pairs_processed**	368,159,700	397,151,443	765,311,143	302,731,332	363,609,258	666,340,590
**Unmapped_pairs**	4,879,653	14,151,080	19,030,733	4,342,040	12,816,925	17,158,965
**Low_qual_pairs**	78,719,442	109,799,587	188,519,029	63,962,532	92,764,907	156,727,439
**Unique_paired_alignments**	264,984,204	260,913,167	525,897,371	218,556,473	245,237,689	463,794,162
**Multiple_pairs_alignments**	0	0	0	0	0	0
**Pairs_with_singleton**	19,576,401	12,287,609	31,864,010	15,870,287	12,789,737	28,660,024
**Low_qual_singleton**	0	0	0	0	0	0
**Unique_singleton_alignments**	0	0	0	0	0	0
**Multiple_singleton_alignments**	0	0	0	0	0	0
**Reported_pairs**	264,984,204	260,913,167	525,897,371	218,556,473	245,237,689	463,794,162
**valid_interaction**	240,198,380	252,429,133	492,627,513	193,097,152	228,799,012	421,896,164
**valid_interaction_rmdup**	208,703,342	208,151,620	416,832,838	169,268,674	193,205,516	362,456,786
**trans_interaction**	44,531,743	44,286,357	88,813,781	32,456,693	37,107,416	69,561,278
**cis_interaction**	164,171,599	163,865,263	328,019,057	136,811,981	156,098,100	292,895,508
**cis_shortRange**	25,447,290	25,529,876	50,973,138	23,769,443	26,294,301	50,059,437
**cis_longRange**	138,724,309	138,335,387	277,045,919	113,042,538	129,803,799	242,836,071
	**KO rep1**	**KO rep2**	**Merge KO**	**KOL rep1**	**KOL rep2**	**Merge KOL**
**Total_pairs_processed**	342,530,432	369,339,350	711,869,782	291,086,961	362,786,944	653,873,905
**Unmapped_pairs**	6,480,715	14,479,603	20,960,318	6,613,439	17,437,801	24,051,240
**Low_qual_pairs**	74,981,170	95,913,993	170,895,163	64,939,888	93,199,208	158,139,096
**Unique_paired_alignments**	239,337,451	243,144,876	482,482,327	200,191,531	235,590,810	435,782,341
**Multiple_pairs_alignments**	0	0	0	0	0	0
**Pairs_with_singleton**	21,731,096	15,800,878	37,531,974	19,342,103	16,559,125	35,901,228
**Low_qual_singleton**	0	0	0	0	0	0
**Unique_singleton_alignments**	0	0	0	0	0	0
**Multiple_singleton_alignments**	0	0	0	0	0	0
**Reported_pairs**	239,337,451	243,144,876	482,482,327	200,191,531	235,590,810	435,782,341
**valid_interaction**	213,221,682	227,939,397	441,161,079	181,464,435	219,663,365	401,127,800
**valid_interaction_rmdup**	185,303,734	192,994,508	378,281,077	161,040,008	185,555,879	346,582,069
**trans_interaction**	41,139,543	41,818,649	82,955,085	41,185,964	45,618,865	86,801,735
**cis_interaction**	144,164,191	151,175,859	295,325,992	119,854,044	139,937,014	259,780,334
**cis_shortRange**	23,802,376	24,799,903	48,598,270	18,345,856	21,944,059	40,286,991
**cis_longRange**	120,361,815	126,375,956	246,727,722	101,508,188	117,992,955	219,493,343

	WT rep3	WT rep4	Merge WT (3-4)	WT+JSH23 rep1	WT+JSH23 rep2	Merge WT+JSH23
**Total_pairs_processed**	400,880,908	413,555,330	814,436,238	395,243,169	412,709,781	807,952,950
**Unmapped_pairs**	4,149,359	4,311,947	8,461,306	3,867,048	4,335,449	8,202,497
**Low_qual_pairs**	99,942,445	103,156,799	203,099,244	99,450,403	103,523,622	202,974,025
**Unique_paired_alignments**	274,321,444	283,957,211	558,278,655	272,380,548	281,933,180	554,313,728
**Multiple_pairs_alignments**	0	0	0	0	0	0
**Pairs_with_singleton**	22,467,660	22,129,373	44,597,033	19,545,170	22,917,530	42,462,700
**Low_qual_singleton**	0	0	0	0	0	0
**Unique_singleton_alignments**	0	0	0	0	0	0
**Multiple_singleton_alignments**	0	0	0	0	0	0
**Reported_pairs**	274,321,444	283,957,211	558,278,655	272,380,548	281,933,180	554,313,728
**valid_interaction**	245,882,830	254,594,655	500,477,485	248,962,365	254,966,748	503,929,113
**valid_interaction_rmdup**	217,258,034	226,456,450	443,705,837	206,102,959	221,653,155	427,748,666
**trans_interaction**	39,130,443	40,819,798	79,949,728	37,936,661	40,954,596	78,890,781
**cis_interaction**	178,127,591	185,636,652	363,756,109	168,166,298	180,698,559	348,857,885
**cis_shortRange**	38,291,359	40,031,552	78,315,707	34,429,278	36,376,890	70,800,143
**cis_longRange**	139,836,232	145,605,100	285,440,402	133,737,020	144,321,669	278,057,742

	WTL rep3	WTL rep4	Merge WTL (3-4)	WTL+JSH23 rep1	WTL+JSH23 rep2	Merge WTL+JSH23
**Total_pairs_processed**	361,334,631	395,880,245	757,214,876	375,762,906	370,882,048	746,644,954
**Unmapped_pairs**	3,606,984	3,479,781	7,086,765	3,425,150	3,117,932	6,543,082
**Low_qual_pairs**	90,827,551	99,571,872	190,399,423	96,225,663	94,253,571	190,479,234
**Unique_paired_alignments**	248,846,601	272,166,583	521,013,184	258,870,214	256,361,858	515,232,072
**Multiple_pairs_alignments**	0	0	0	0	0	0
**Pairs_with_singleton**	18,053,495	20,662,009	38,715,504	17,241,879	17,148,687	34,390,566
**Low_qual_singleton**	0	0	0	0	0	0
**Unique_singleton_alignments**	0	0	0	0	0	0
**Multiple_singleton_alignments**	0	0	0	0	0	0
**Reported_pairs**	248,846,601	272,166,583	521,013,184	258,870,214	256,361,858	515,232,072
**valid_interaction**	223,904,087	246,059,544	469,963,631	238,965,839	236,438,520	475,404,359
**valid_interaction_rmdup**	197,720,420	216,513,112	414,226,551	196,937,431	199,578,259	396,509,852
**trans_interaction**	34,600,399	37,854,452	72,454,485	36,265,849	36,671,210	72,936,742
**cis_interaction**	163,120,021	178,658,660	341,772,066	160,671,582	162,907,049	323,573,110
**cis_shortRange**	34,791,921	38,043,481	72,829,568	31,847,866	32,124,140	63,967,236
**cis_longRange**	128,328,100	140,615,179	268,942,498	128,823,716	130,782,909	259,605,874

Supplementary Table 4. Summary of RelA HiChIP

	WTL rep1	WTL rep2	Merge WTL
Total_pairs_processed	339,935,652	333,946,591	673,882,243
Unmapped_pairs	25,139,401	3,388,656	28,528,062
Low_qual_pairs	105,534,694	78,630,962	184,165,676
Unique_paired_alignments	191,057,910	238,198,194	429,256,099
Multiple_pairs_alignments	0	0	0
Pairs_with_singleton	18,203,647	13,728,779	31,932,406
Low_qual_singleton	0	0	0
Unique_singleton_alignments	0	0	0
Multiple_singleton_alignments	0	0	0
Reported_pairs	191,057,910	238,198,194	429,256,099
valid_interaction	165,372,818	205,450,117	370,822,931
valid_interaction_rmdup	63,045,488	105,064,931	168,110,416
trans_interaction	13,401,685	17,344,882	30,746,564
cis_interaction	49,643,803	87,720,049	137,363,852
cis_shortRange	10,823,047	21,175,149	31,998,196
cis_longRange	38,820,756	66,544,900	105,365,656

Supplementary Table 5. Upregulated genes related to Figure 4f

Differential analyses were calculated using the two-sided Wald test by the nbinomWaldTest function in DESeq2.

gene	baseMean	Log2FC	padj	WT rep1	WT rep2	WT rep3	KO rep1	KO rep2	KO rep3
GM14964	3.03	5.10	2.56.E-03	0.00	0.00	0.00	2.11	0.36	1.42
CD69	4015.43	4.04	0.00.E+00	31.87	32.48	33.21	552.55	516.16	556.84
ALDH1A2	3597.88	3.07	0.00.E+00	33.77	32.69	31.44	261.41	261.96	241.26
CLEC1A	152.32	3.01	5.13.E-53	0.78	0.89	0.55	6.24	5.73	5.14
NDST1	2242.21	2.96	0.00.E+00	10.24	11.12	10.18	74.50	72.84	75.43
ATG3	3021.59	2.17	0.00.E+00	52.11	52.64	51.40	218.98	215.44	219.60
SCN2B	54.69	2.08	7.78.E-13	0.53	0.45	0.33	1.82	1.82	1.41
SUSD1	223.86	1.84	1.77.E-40	3.29	4.37	5.94	17.44	15.79	18.18
CCNB2-PS	25.10	1.84	5.09.E-05	0.65	1.41	0.48	1.98	1.64	2.40
ZFP882	65.49	1.84	7.57.E-13	1.15	1.70	1.05	2.89	3.47	3.00
CDC42BPG	847.68	1.78	7.00.E-127	6.22	6.39	5.49	21.74	22.69	19.83
SLC35A5	968.11	1.78	3.08.E-139	11.54	9.27	11.54	37.14	34.53	37.39
FCGR4	115.34	1.71	7.60.E-19	3.79	5.28	4.56	13.97	13.51	14.16
SPACA6	383.96	1.69	2.74.E-46	16.19	11.54	15.72	34.03	36.63	46.60
KAZN	75.74	1.60	1.16.E-11	0.94	0.85	1.00	2.74	2.35	3.57
ALYREF2	127.09	1.58	2.90.E-18	4.56	6.02	5.19	14.82	15.25	14.08
CDO1	8.96	1.58	4.77.E-02	0.26	0.32	0.30	1.55	1.45	0.40
NMB	84.41	1.53	3.13.E-11	5.57	9.29	9.12	21.58	22.39	22.03
CD48	2420.13	1.46	1.96.E-183	118.66	112.16	120.17	294.17	299.88	306.05
KCTD12	13414.04	1.44	1.57.E-215	116.63	98.37	108.05	264.81	276.93	273.19
GM44243	14.83	1.41	1.67.E-02	2.57	1.38	0.69	4.14	3.00	4.10
GM44154	80.76	1.40	3.60.E-10	1.48	1.73	1.40	3.51	3.93	3.94
SFMBT1	834.19	1.37	2.21.E-73	6.64	7.67	7.10	16.13	15.55	13.68
PDXK	1474.87	1.36	3.40.E-113	13.90	15.10	14.76	34.92	34.56	37.26
EEA1	2722.09	1.36	7.86.E-176	19.45	17.86	19.32	44.60	45.56	45.06
ZBTB10	88.59	1.31	2.32.E-09	0.50	0.68	0.66	1.53	1.47	1.25
SFXN2	459.02	1.31	1.81.E-38	6.63	7.87	7.53	19.09	18.11	15.01
GABARAPL1	6222.85	1.31	2.57.E-265	259.99	245.96	246.43	620.72	629.80	608.09
IL1B	3024.10	1.30	1.06.E-171	149.37	142.66	139.70	338.60	348.53	323.70
ALOX15	12.04	1.28	4.71.E-02	0.37	0.37	0.26	0.62	0.73	0.80
RASSF4	18567.69	1.26	0.00.E+00	188.07	194.98	219.35	434.72	451.09	458.03
ATF6	2105.24	1.24	3.82.E-133	15.62	15.43	15.55	34.34	33.59	34.24
GM37010	15.26	1.19	3.16.E-02	0.49	0.49	0.66	1.19	1.12	1.18
MED11	632.77	1.18	2.56.E-44	46.19	53.95	50.21	105.53	107.89	105.81
PEX5	869.96	1.17	1.23.E-59	19.06	18.30	18.58	38.22	39.77	37.28
RAC2	6791.39	1.15	3.39.E-160	147.37	187.55	156.63	289.25	298.71	300.82
SLC22A21	197.24	1.11	3.78.E-14	6.29	5.04	4.89	12.50	11.18	10.87
HIST1H1C	1777.20	1.09	6.82.E-92	73.26	76.18	71.88	147.83	147.16	145.11
SNX29	96.52	1.08	1.78.E-07	2.28	2.67	2.88	4.76	4.34	4.15
GM20517	27.41	1.05	2.43.E-02	3.52	1.83	1.59	2.69	5.56	5.39
KLHL20	721.77	1.05	2.28.E-41	13.08	11.73	12.14	22.22	25.22	23.35
AFMID	175.52	1.03	6.47.E-12	6.66	7.08	6.24	11.52	10.65	13.71
SYNGR1	192.77	1.03	3.39.E-12	13.09	13.92	12.12	16.72	17.62	18.09
PILRB1	693.06	0.97	2.73.E-36	67.23	55.34	53.40	105.18	110.14	109.23
LNPEP	6825.30	0.94	1.36.E-88	37.67	32.58	36.34	62.67	64.74	62.12
SPATS2	56.92	0.94	5.50.E-04	1.28	1.54	1.50	3.38	2.42	2.28
ENTPD1	1839.61	0.93	1.43.E-67	42.37	44.00	50.23	81.43	88.49	76.23
FAHD1	75.31	0.91	8.89.E-05	3.41	3.53	3.97	6.53	6.55	6.05
TREML2	274.57	0.90	3.65.E-13	4.49	5.23	4.67	8.16	9.83	8.28
SLC16A13	83.58	0.89	1.12.E-04	4.80	3.09	6.86	5.77	9.66	7.63
FCHO1	836.05	0.86	3.95.E-32	66.49	54.59	61.91	89.02	100.95	98.39
MED14	1362.42	0.86	1.07.E-43	14.06	12.89	14.35	22.36	22.78	24.56
IRGQ	355.58	0.85	8.68.E-15	3.66	4.13	3.48	5.93	6.60	6.37
ST3GAL1	5017.42	0.84	3.55.E-77	60.45	64.09	64.68	102.58	108.19	100.92
CLN5	1951.87	0.82	1.87.E-51	56.61	56.00	57.12	92.00	91.96	93.47
HK2	1644.07	0.81	1.36.E-10	31.22	40.77	37.82	55.11	50.67	51.26
FOXP4	878.04	0.79	6.97.E-29	18.30	22.58	18.12	29.78	35.18	33.16
COX5A	1785.34	0.79	6.23.E-45	297.56	261.47	246.86	438.24	425.42	427.45
TCTN3	206.71	0.77	4.44.E-08	4.85	5.70	5.53	8.89	10.12	8.83
WDFY2	1509.45	0.74	4.73.E-41	15.55	15.22	15.51	24.09	24.70	24.12

SIRPA	14142.21	0.72	3.27.E-100	349.51	312.89	302.46	535.23	553.40	561.58
RPS6KA4	1131.61	0.72	2.63.E-26	32.02	32.02	30.86	51.35	50.26	56.34
IFI30	9452.75	0.71	5.18.E-70	807.81	862.23	803.21	1244.76	1277.99	1259.04
CACFD1	310.33	0.71	9.01.E-10	11.29	11.04	11.77	17.30	18.13	19.70
UNC13A	183.77	0.71	1.59.E-05	1.35	1.53	1.04	2.03	1.73	2.15
CD84	4823.24	0.71	7.50.E-61	155.29	148.21	154.56	234.30	231.88	232.45
GM21988	154.34	0.70	9.16.E-05	6.10	6.66	8.07	8.71	11.62	11.60
TFEC	1499.29	0.70	4.76.E-35	64.46	62.70	64.96	97.09	94.23	97.23
ABHD8	63.05	0.70	1.09.E-02	3.15	2.19	2.17	3.95	3.46	3.33
RNASEK	1105.33	0.70	5.03.E-26	207.47	178.33	185.12	292.09	281.98	291.17
PRDX5	10373.52	0.69	7.77.E-87	1206.20	1262.50	1215.40	1946.90	1996.04	1966.23
MRPS28	119.21	0.68	2.49.E-04	14.32	16.00	12.55	21.47	22.24	20.58
JDP2	96.01	0.68	1.74.E-03	4.77	5.29	4.07	8.03	6.59	6.94
NELFE	445.26	0.67	3.82.E-11	27.48	27.39	24.48	38.67	43.07	43.42
NRIP1	910.25	0.67	1.11.E-21	8.18	8.78	9.20	13.49	12.83	12.07
MESD	756.06	0.66	1.43.E-18	15.47	12.38	14.85	20.08	20.97	19.11
CCDC71L	1007.38	0.66	3.29.E-17	19.17	15.03	16.49	25.09	25.59	23.70
MPC2	354.90	0.63	3.22.E-09	38.46	35.04	33.18	46.17	47.24	39.79
KCNA3	207.17	0.63	7.43.E-06	8.29	8.25	8.68	11.43	11.82	13.19
ZFP422	439.58	0.63	6.84.E-10	13.81	11.01	11.14	16.46	18.49	18.40
ARPIN	217.05	0.62	5.99.E-06	7.38	6.93	7.41	10.66	10.71	9.69
STIMATE	245.33	0.61	2.45.E-06	9.14	7.01	9.83	12.35	10.03	11.88
TXN2	1555.25	0.61	6.10.E-23	101.02	109.20	93.33	141.13	144.34	142.91
AHNAK	27674.93	0.60	6.46.E-82	127.61	129.89	125.03	168.37	170.76	165.85
RCAN1	295.38	0.60	2.53.E-07	10.15	9.99	10.61	14.18	14.47	14.28
ARL3	304.63	0.58	1.23.E-06	33.66	35.19	33.10	44.07	54.39	45.40
NDUFA10	1077.13	0.57	2.12.E-16	48.48	50.54	44.70	62.13	65.74	70.53
COPA	4746.32	0.57	4.13.E-39	80.92	89.87	83.06	114.33	115.90	121.52
ISCU	771.45	0.57	1.70.E-13	71.26	80.88	68.02	99.21	102.10	100.90
CEP95	162.97	0.57	6.19.E-04	7.26	8.73	6.43	8.73	9.11	10.22
PTPRC	19818.32	0.57	1.08.E-63	334.99	309.24	318.43	448.85	455.34	457.99
ZCWPW1	81.77	0.57	1.38.E-02	5.09	4.56	3.48	5.78	6.55	6.78
IFT43	107.02	0.57	9.75.E-03	16.03	15.03	10.47	21.33	15.72	19.65
PIRB	6514.90	0.57	9.11.E-47	155.57	159.56	152.63	206.61	216.35	220.14
GM44423	112.96	0.57	3.57.E-03	2.82	2.59	2.67	3.37	4.05	3.73
MPV17L2	164.30	0.56	4.64.E-04	15.73	20.41	16.98	25.03	23.56	23.81
SMPDL3A	1689.41	0.56	8.57.E-24	72.48	69.40	64.47	93.34	94.83	96.62
ZFP799	166.38	0.56	1.04.E-03	2.14	2.30	4.32	2.34	2.99	4.54
MEPCE	628.88	0.55	4.36.E-11	18.63	19.86	21.16	25.84	28.76	28.07
MAP4K1	1181.47	0.53	3.75.E-17	42.45	37.19	39.79	46.81	59.07	48.11
PCK2	312.47	0.53	2.90.E-06	9.77	9.40	10.65	14.18	12.37	11.89
ARL6IP5	2909.70	0.52	3.54.E-28	171.16	169.37	171.41	222.81	225.11	236.15
RAB43	2714.72	0.51	8.00.E-22	45.14	48.60	50.23	62.25	64.58	65.99
LRCH1	511.26	0.50	2.51.E-07	10.25	11.33	9.37	13.66	13.66	12.38
PILRA	2568.33	0.50	2.47.E-24	144.74	152.69	171.98	193.39	182.73	197.68
SLC25A11	1038.10	0.50	2.15.E-14	62.38	66.71	59.91	78.16	82.77	81.38
ALDH18A1	281.66	0.48	9.89.E-05	8.11	5.93	6.81	7.36	11.37	8.33
MED22	1072.98	0.47	3.28.E-13	24.82	25.12	24.88	30.56	33.37	31.52
GM44103	184.06	0.47	3.12.E-03	4.36	3.90	3.41	4.48	5.49	5.12
COA5	2255.51	0.46	1.82.E-20	44.51	46.24	45.16	58.91	58.58	57.82
PLXNC1	2108.95	0.46	4.46.E-18	22.71	24.29	26.13	29.38	30.40	26.70
EME2	248.72	0.46	4.16.E-04	7.17	7.05	7.37	9.16	9.34	11.87
CTSB	25071.85	0.44	7.12.E-29	511.83	523.24	526.87	587.34	593.69	596.76
TMEM51	344.06	0.43	2.50.E-04	16.53	16.52	13.25	18.64	20.28	19.18
EMC10	665.22	0.43	2.19.E-07	35.11	35.63	34.26	43.99	48.45	41.80
TLNRD1	395.42	0.42	6.39.E-05	7.56	7.04	6.30	8.92	8.62	8.53
ITGB7	1039.09	0.42	1.95.E-10	54.01	47.15	47.30	56.98	59.32	56.40
CSTB	8435.50	0.42	5.91.E-33	1715.85	1682.11	1611.22	2086.58	2079.15	2081.39
TMEM106B	1115.95	0.42	2.44.E-10	15.25	16.72	14.37	25.43	21.71	19.82
LAIR1	630.97	0.41	3.15.E-06	21.96	15.64	16.63	21.07	21.28	22.02
TMEM170B	1378.41	0.40	6.24.E-10	16.19	13.90	14.92	18.66	19.25	19.22
UBXN2B	140.47	0.39	2.88.E-02	1.59	1.65	1.67	1.92	1.92	2.18
TRMT1	481.89	0.39	5.82.E-05	30.42	34.97	31.52	36.17	39.98	35.40
