# Supplementary material for: CTCF controls three-dimensional enhancer network underlying the inflammatory response of bone marrow-derived dendritic cells
Source: Nat Commun. 2023 Mar 8;14:1277. doi: 10.1038/s41467-023-36948-5 (PMC9992691; doi:10.1038/s41467-023-36948-5)
Supplement: Supplementary file 3 — Reporting Summary [file 41467_2023_36948_MOESM3_ESM.pdf]

nature portfolio

Corresponding author(s): Hyoung-Pyo Kim

Last updated by author(s): Jan 2, 2023

Reporting Summary

Nature Portfolio wishes to improve the reproducibility of the work that we publish. This form provides structure for consistency and transparency in reporting. For further information on Nature Portfolio policies, see our Editorial Policies and the Editorial Policy Checklist.

Statistics

For all statistical analyses, confirm that the following items are present in the figure legend, table legend, main text, or Methods section.

n/a	Confirmed	
☐	☒	The exact sample size (*n*) for each experimental group/condition, given as a discrete number and unit of measurement
☐	☒	A statement on whether measurements were taken from distinct samples or whether the same sample was measured repeatedly
☐	☒	The statistical test(s) used AND whether they are one- or two-sided<br>*Only common tests should be described solely by name; describe more complex techniques in the Methods section.*
☒	☐	A description of all covariates tested
☐	☒	A description of any assumptions or corrections, such as tests of normality and adjustment for multiple comparisons
☐	☒	A full description of the statistical parameters including central tendency (e.g. means) or other basic estimates (e.g. regression coefficient) AND variation (e.g. standard deviation) or associated estimates of uncertainty (e.g. confidence intervals)
☐	☒	For null hypothesis testing, the test statistic (e.g. *F*, *t*, *r*) with confidence intervals, effect sizes, degrees of freedom and *P* value noted<br>*Give P values as exact values whenever suitable.*
☒	☐	For Bayesian analysis, information on the choice of priors and Markov chain Monte Carlo settings
☒	☐	For hierarchical and complex designs, identification of the appropriate level for tests and full reporting of outcomes
☒	☐	Estimates of effect sizes (e.g. Cohen's *d*, Pearson's *r*), indicating how they were calculated

Our web collection on statistics for biologists contains articles on many of the points above.

Software and code

Policy information about availability of computer code

Data collection	Flow cytometry: BD FACSuiteTM v1.0.6 and FACSDivaTM v8.0.2<br>RT-PCR QuantStudio 3: QuantStudioTM Design & Analysis Software v1.4.1<br>Confocal imaging: ZEN 2012 v1.1.2.0<br>Western blotting: ImageQuant LAS 4000 Control Software v1.2.1.119<br>Illumina Next-generation sequencing
Data analysis	Flow cytometry analysis: FlowJo(v10.5.3), BDTM Cytometric Bead Array FCAP Array Software(v3.0)<br>GraphPad Prism(v5.03)<br>Imaging analysis: ImageJ software(v1.52)<br>RNA-seq: Fastqc(v0.11.5), Trim Galore(v0.6.4), STAR(v2.6.0a), RSEM(v1.2.31), DEseq2(v1.24.0)<br>ChIP-seq: Fastqc(v0.11.5), Trim Galore(v0.6.4), bwa(v0.7.17), picard(v2.9.2), MACS2(v2.2.7.1), deepTools(v3.3.0), IGV(v2.8.2)<br>Hi-C: HiC-Pro(v2.11.1), HiCSpector(run_reproducibility_v2.py), Juicebox(v1.9.8), Juicer tools(v1.19.02)<br>HiChIP: HiC-Pro(v2.11.1), HiCSpector(run_reproducibility_v2.py), Juicebox(v1.9.8), FitHiChIP(v6.0), DESeq2(v1.24.0), LOLA(v1.8.0), ROSE(v0.1), igraph R package(v1.3.2)

For manuscripts utilizing custom algorithms or software that are central to the research but not yet described in published literature, software must be made available to editors and reviewers. We strongly encourage code deposition in a community repository (e.g. GitHub). See the Nature Portfolio guidelines for submitting code & software for further information.

Data

Policy information about availability of data

All manuscripts must include a data availability statement. This statement should provide the following information, where applicable:

- Accession codes, unique identifiers, or web links for publicly available datasets
- A description of any restrictions on data availability
- For clinical datasets or third party data, please ensure that the statement adheres to our policy

All newly-generated RNA-seq, ChIP-seq, in situ Hi-C, and HiChIP data have been deposited in the Gene Expression Omnibus (GEO) under accession number GSE185884 (https://www.ncbi.nlm.nih.gov/geo/query/acc.cgi?acc=GSE185884).
Figure 1: ChIP-seq (CTCF, SMC1)
Figure 2: Hi-C, H3K27ac HiChIP
Figure 3: H3K27ac ChIP-seq, H3K27ac HiChIP, RNA-seq
Figure 4: RNA-seq, Hi-C, H3K27ac HiChIP, ChIP-seq (H3K27ac, H3K4me3, H3K27me3, H3K4me1, CTCF, SMC1)
Figure 5: RNA-seq, H3K27ac HiChIP, ChIP-seq (H3K27ac, H3K4me3, H3K27me3, H3K4me1, STAT5, CTCF, SMC1)
Figure 6: ChIP-seq (RelA, SMC1, H3K27ac), H3K27ac HiChIP
Figure 7: RNA-seq, HiChIP (RelA, H3K27ac), ChIP-seq (RelA, CTCF, SMC1, H3K27ac)
Figure 8: RNA-seq, H3K27ac HiChIP, H3K27ac ChIP-seq
Supplementary Figure 2: ChIP-seq (CTCF, SMC1)
Supplementary Figure 4: Hi-C, CTCF ChIP-seq
Supplementary Figure 5: H3K27ac HiChIP, ChIP-seq (CTCF, SMC1)
Supplementary Figure 6: RNA-seq, H3K27ac HiChIP, ChIP-seq (H3K27ac, H3K4me3, H3K27me3, H3K4me1, CTCF, SMC1)
Supplementary Figure 9: RNA-seq, RelA HiChIP, RelA ChIP-seq
Supplementary Figure 10: HiChIP (RelA, H3K27ac)

Human research participants

Policy information about studies involving human research participants and Sex and Gender in Research.

Reporting on sex and gender	n/a
Population characteristics	n/a
Recruitment	n/a
Ethics oversight	n/a

Note that full information on the approval of the study protocol must also be provided in the manuscript.

Field-specific reporting

Please select the one below that is the best fit for your research. If you are not sure, read the appropriate sections before making your selection.

☒ Life sciences ☐ Behavioural & social sciences ☐ Ecological, evolutionary & environmental sciences

For a reference copy of the document with all sections, see nature.com/documents/nr-reporting-summary-flat.pdf

Life sciences study design

All studies must disclose on these points even when the disclosure is negative.

Sample size	No sample size calculations were performed and number of replicates were based on standard practices in the field. Sample size were indicated in the figure legends
Data exclusions	No data were excluded.
Replication	Experiments were performed with 2-3 biological replicates for each condition. After confirmation of good reproducibility, we merged the replicates and re-processed the data as combined results.<br>All attempts at replication were successful.
Randomization	Randomization was not used in this study. All samples were prepared from mouse primary cell, the groups were determined by genotype.
Blinding	Blinding of samples was not performed in this study since our samples had obvious phenotypes depending on the genotype and the results were consistent.

Behavioural & social sciences study design

All studies must disclose on these points even when the disclosure is negative.

Study description	*Briefly describe the study type including whether data are quantitative, qualitative, or mixed-methods (e.g. qualitative cross-sectional, quantitative experimental, mixed-methods case study).*
Research sample	*State the research sample (e.g. Harvard university undergraduates, villagers in rural India) and provide relevant demographic information (e.g. age, sex) and indicate whether the sample is representative. Provide a rationale for the study sample chosen. For studies involving existing datasets, please describe the dataset and source.*
Sampling strategy	*Describe the sampling procedure (e.g. random, snowball, stratified, convenience). Describe the statistical methods that were used to predetermine sample size OR if no sample-size calculation was performed, describe how sample sizes were chosen and provide a rationale for why these sample sizes are sufficient. For qualitative data, please indicate whether data saturation was considered, and what criteria were used to decide that no further sampling was needed.*
Data collection	*Provide details about the data collection procedure, including the instruments or devices used to record the data (e.g. pen and paper, computer, eye tracker, video or audio equipment) whether anyone was present besides the participant(s) and the researcher, and whether the researcher was blind to experimental condition and/or the study hypothesis during data collection.*
Timing	*Indicate the start and stop dates of data collection. If there is a gap between collection periods, state the dates for each sample cohort.*
Data exclusions	*If no data were excluded from the analyses, state so OR if data were excluded, provide the exact number of exclusions and the rationale behind them, indicating whether exclusion criteria were pre-established.*
Non-participation	*State how many participants dropped out/declined participation and the reason(s) given OR provide response rate OR state that no participants dropped out/declined participation.*
Randomization	*If participants were not allocated into experimental groups, state so OR describe how participants were allocated to groups, and if allocation was not random, describe how covariates were controlled.*

Ecological, evolutionary & environmental sciences study design

All studies must disclose on these points even when the disclosure is negative.

Study description	*Briefly describe the study. For quantitative data include treatment factors and interactions, design structure (e.g. factorial, nested, hierarchical), nature and number of experimental units and replicates.*
Research sample	*Describe the research sample (e.g. a group of tagged Passer domesticus, all Stenocereus thurberi within Organ Pipe Cactus National Monument), and provide a rationale for the sample choice. When relevant, describe the organism taxa, source, sex, age range and any manipulations. State what population the sample is meant to represent when applicable. For studies involving existing datasets, describe the data and its source.*
Sampling strategy	*Note the sampling procedure. Describe the statistical methods that were used to predetermine sample size OR if no sample-size calculation was performed, describe how sample sizes were chosen and provide a rationale for why these sample sizes are sufficient.*
Data collection	*Describe the data collection procedure, including who recorded the data and how.*
Timing and spatial scale	*Indicate the start and stop dates of data collection, noting the frequency and periodicity of sampling and providing a rationale for these choices. If there is a gap between collection periods, state the dates for each sample cohort. Specify the spatial scale from which the data are taken*
Data exclusions	*If no data were excluded from the analyses, state so OR if data were excluded, describe the exclusions and the rationale behind them, indicating whether exclusion criteria were pre-established.*
Reproducibility	*Describe the measures taken to verify the reproducibility of experimental findings. For each experiment, note whether any attempts to repeat the experiment failed OR state that all attempts to repeat the experiment were successful.*
Randomization	*Describe how samples/organisms/participants were allocated into groups. If allocation was not random, describe how covariates were controlled. If this is not relevant to your study, explain why.*
Blinding	*Describe the extent of blinding used during data acquisition and analysis. If blinding was not possible, describe why OR explain why blinding was not relevant to your study.*

Did the study involve field work? ☐ Yes ☐ No

Field work, collection and transport

Field conditions	*Describe the study conditions for field work, providing relevant parameters (e.g. temperature, rainfall).*
Location	*State the location of the sampling or experiment, providing relevant parameters (e.g. latitude and longitude, elevation, water depth).*
Access & import/export	*Describe the efforts you have made to access habitats and to collect and import/export your samples in a responsible manner and in compliance with local, national and international laws, noting any permits that were obtained (give the name of the issuing authority, the date of issue, and any identifying information).*
Disturbance	*Describe any disturbance caused by the study and how it was minimized.*

Reporting for specific materials, systems and methods

We require information from authors about some types of materials, experimental systems and methods used in many studies. Here, indicate whether each material, system or method listed is relevant to your study. If you are not sure if a list item applies to your research, read the appropriate section before selecting a response.

Materials & experimental systems

n/a	Involved in the study	
☐	☒	Antibodies
☐	☒	Eukaryotic cell lines
☒	☐	Palaeontology and archaeology
☐	☒	Animals and other organisms
☒	☐	Clinical data
☒	☐	Dual use research of concern

Methods

n/a	Involved in the study	
☐	☒	ChIP-seq
☐	☒	Flow cytometry
☒	☐	MRI-based neuroimaging

Antibodies

Antibodies used

For ChIP-seq and HiChIP: 1μg for histones, 5μg for transcription factors
For western blot: 1:1000 dilution for primary antibodies, 1:2000 dilution for secondary antibodies
For flow cytometry: 1:200 dilution
For immunofluorescence assay: 1:100 dilution
Amount/dilution of antibodies were indicated in Methods section.
anti-H3K27ac (ChIP-seq, HiChIP): Abcam ab4729, lot GR3251519-1
anti-H3K27me3 (ChIP-seq): Abcam ab6002, lot GR3306698-2
anti-H3K4me1 (ChIP-seq): Abcam ab8895, lot GR3264996-2
anti-H3K4me3 (ChIP-seq): Abcam ab8580, lot GR288375-1
anti-SMC1 (ChIP-seq): Bethyl lab A300-055A, lot 6
anti-CTCF (western blotting, ChIP-seq): Cell Signaling Technology 2899, lot 2
anti-NF-κB p65 (western blotting, ChIP-seq, HiChIP, IF assay): Cell Signaling Technology 8242, lot 13
anti-β-actin (western blotting): Santa Cruz sc-47778, lot K0216
anti-Phospho-IKKα/β (western blotting): Cell Signaling Technology 2078, lot 9
anti-IKKα/β (western blotting): Cell Signaling Technology 2682, lot 5
anti-Phospho-IκBα (western blotting): Cell Signaling Technology 2859, lot 17
anti-IκBα (western blotting): Cell Signaling Technology 9242, lot 10
anti-Lamin B1 (western blotting): Abcam ab133741, lot GR3233597-3
anti-Phospho-JAK2 (western blotting): Cell Signaling Technology 3771, lot 10
anti-JAK2 (western blotting): Cell Signaling Technology 3230, lot 11
anti-Phospho-STAT5 (western blotting): Cell Signaling Technology 9351, lot 9
anti-STAT5 (western blotting, ChIP-seq): Cell Signaling Technology 94205, lot 3
anti-α-Tubulin (western blotting): Santa Cruz sc-32293, lot I2413
anti-Aldh1a2 (western blotting): Abcam ab156019, lot YJ103114CS
HRP-linked anti-Rabbit IgG (western blotting): Cell Signaling Technology 7074, lot 28
HRP-linked anti-Mouse IgG (western blotting): Cell Signaling Technology 7076, lot 34
Goat anti-Rabbit IgG (H+L) Secondary Antibody, Alexa Fluor 594 (Immunofluorescence assay): Invitrogen A-11037
anti-CD11c (PerCP-Cy5.5) (Flow cytometric analysis): eBioscience 45-0114-82, lot E08306-1633
anti-CD11b (APC-eFluor780) (Flow cytometric analysis): eBioscience 47-0112-82, lot E10339-1633
anti-CD11b (FITC) (Flow cytometric analysis): eBioscienc 11-0112-85, lot E00149-160
anti-CD4 (FITC) (Flow cytometric analysis): eBioscience 11-0042-82, lot E00083-1630
anti-IFNγ (PerCP-Cy5.5) (Flow cytometric analysis): eBioscience 45-7311-80, lot 4329723

anti-IL-17A (APC) (Flow cytometric analysis): eBioscience 17-7177-81, lot 1936926
anti-Annexin V/PI kit (Flow cytometric analysis): eBioscience 88-8007-74
anti-I-A/E (PE) (Flow cytometric analysis): eBioscience 12-5321-82, lot E01733-1631
anti-I-A/E (APC-Cy7) (Flow cytometric analysis): eBioscience 47-5321-82, lot E10058-1632
anti-CD80 (APC) (Flow cytometric analysis): eBioscience 17-0801-82, lot E07180-1373
anti-CD86 (FITC) (Flow cytometric analysis): eBioscience 11-0862-82, lot E00420-1630
anti-CD45.1 (PE) (Flow cytometric analysis): eBioscience 12-0453-83
anti-CD45.2 (APC-eFluor780) (Flow cytometric analysis): eBioscience 47-0454-82, lot E08455-1639
anti-CD135 (PE) (Flow cytometric analysis): eBioscience 12-1351-82, lot E01494-1634
anti-CD115 (BV421) (Flow cytometric analysis): BD 743638, lot 2080558

Validation

For commercially available antibodies, validation was performed by the manufacturer.
anti-H3K27ac (ChIP-seq, HiChIP): Abcam ab4729, lot GR3251519-1. Validated ChIP in human cell lines on the manufacturer's website
anti-H3K27me3 (ChIP-seq): Abcam ab6002, lot GR3306698-2. Validated ChIP in human cell lines on the manufacturer's website
anti-H3K4me1 (ChIP-seq): Abcam ab8895, lot GR3264996-2. Validated ChIP in human cell lines on the manufacturer's website
anti-H3K4me3 (ChIP-seq): Abcam ab8580, lot GR288375-1. Validated ChIP in human cell lines on the manufacturer's website
anti-SMC1 (ChIP-seq): Bethyl lab A300-055A, lot 6. Validated IP in human cell lines on the manufacturer's website. Validated for ChIP in PMID 28985562
anti-CTCF (western blotting, ChIP-seq): Cell Signaling Technology 2899, lot 2. Validated WB and ChIP in human cell lines on the manufacturer's website
anti-NF-κB p65 (western blotting, ChIP-seq, HiChIP, IF assay): Cell Signaling Technology 8242, lot 13. Validated WB, ChIP, and IF in human cell lines on the manufacturer's website
anti-β-actin (western blotting): Santa Cruz sc-47778, lot K0216. Validated WB in human cell lines on the manufacturer's website
anti-Phospho-IKKα/β (western blotting): Cell Signaling Technology 2078, lot 9. Validated WB in human cell lines on the manufacturer's website
anti-IKKα/β (western blotting): Cell Signaling Technology 2682, lot 5. Validated WB in human cell lines on the manufacturer's website
anti-Phospho-IκBα (western blotting): Cell Signaling Technology 2859, lot 17. Validated WB in human cell lines on the manufacturer's website
anti-IκBα (western blotting): Cell Signaling Technology 9242, lot 10. Validated WB in human cell lines on the manufacturer's website
anti-Lamin B1 (western blotting): Abcam ab133741, lot GR3233597-3. Validated WB in mouse cells on the manufacturer's website
anti-Phospho-JAK2 (western blotting): Cell Signaling Technology 3771, lot 10. Validated WB in human cell lines on the manufacturer's website
anti-JAK2 (western blotting): Cell Signaling Technology 3230, lot 11. Validated WB in human cell lines on the manufacturer's website
anti-Phospho-STAT5 (western blotting): Cell Signaling Technology 9351, lot 9. Validated WB in human cell lines on the manufacturer's website
anti-STAT5 (western blotting, ChIP-seq): Cell Signaling Technology 94205, lot 3. Validated WB and ChIP in mouse cells on the manufacturer's website
anti-α-Tubulin (western blotting): Santa Cruz sc-32293, lot I2413. Validated WB in human cell lines on the manufacturer's website
anti-Aldh1a2 (western blotting): Abcam ab156019, lot YJ103114CS. Validated WB in mouse cells on the manufacturer's website
HRP-linked anti-Rabbit IgG (western blotting): Cell Signaling Technology 7074, lot 28. Validated WB on the manufacturer's website
HRP-linked anti-Mouse IgG (western blotting): Cell Signaling Technology 7076, lot 34. Validated WB on the manufacturer's website
Goat anti-Rabbit IgG (H+L) Secondary Antibody, Alexa Fluor 594 (Immunofluorescence assay): Invitrogen A-11037. Validated IF on the manufacturer's website
anti-CD11c (PerCP-Cy5.5) (Flow cytometric analysis): eBioscience 45-0114-82, lot E08306-1633. Validated Flow Cytometry in mouse cells on the manufacturer's website
anti-CD11b (APC-eFluor780) (Flow cytometric analysis): eBioscience 47-0112-82, lot E10339-1633. Validated Flow Cytometry in mouse cells on the manufacturer's website
anti-CD4 (FITC) (Flow cytometric analysis): eBioscience 11-0042-82, lot E00083-1630. Validated Flow Cytometry in mouse cells on the manufacturer's website
anti-IFNγ (PerCP-Cy5.5) (Flow cytometric analysis): eBioscience 45-7311-80, lot 4329723. Validated Flow Cytometry in mouse cells on the manufacturer's website
anti-IL-17A (APC) (Flow cytometric analysis): eBioscience 17-7177-81, lot 1936926. Validated Flow Cytometry in mouse cells on the manufacturer's website
anti-Annexin V (APC) (Flow cytometric analysis): eBioscience 17-8007-74, lot 2005128. Validated Flow Cytometry in mouse cells on the manufacturer's website
anti-MHC[illegible] (PE) (Flow cytometric analysis): eBioscience 12-5321-82, lot E01733-1631. Validated Flow Cytometry in mouse cells on the manufacturer's website
anti-CD80 (APC) (Flow cytometric analysis): eBioscience 17-0801-82, lot E07180-1373. Validated Flow Cytometry in mouse cells on the manufacturer's website
anti-CD86 (FITC) (Flow cytometric analysis): eBioscience 11-0862-82, lot E00420-1630. Validated Flow Cytometry in mouse cells on the manufacturer's website
anti-CD45.1 (PE) (Flow cytometric analysis): eBioscience 12-0453-83. Validated Flow Cytometry in mouse cells on the manufacturer's website
anti-CD45.2 (APC-eFluor780) (Flow cytometric analysis): eBioscience 47-0454-82, lot E08455-1639. Validated Flow Cytometry in mouse cells on the manufacturer's website
anti-CD135 (PE) (Flow cytometric analysis): eBioscience 12-1351-82, lot E01494-1634. Validated Flow Cytometry in mouse cells on the manufacturer's website
anti-CD115 (BV421) (Flow cytometric analysis): BD 743638, lot 2080558. Validated Flow Cytometry in mouse cells on the manufacturer's website

Eukaryotic cell lines

Policy information about cell lines and Sex and Gender in Research

Cell line source(s)	Human 293FT cell lines were purchased from ATCC.<br>Mouse bone marrow-derived dendritic cells were generated from bone marrow (both genders).
Authentication	Human 293FT cells were authenticated by ATCC. Mouse BMDCs were confirmed to express cell lineage marker by Flow Cytometry for authentication.
Mycoplasma contamination	Cell lines were tested negative for mycoplasma contamination.
Commonly misidentified lines<br>(See ICLAC register)	No commonly misidentified cell lines were used.

Palaeontology and Archaeology

Specimen provenance	*Provide provenance information for specimens and describe permits that were obtained for the work (including the name of the issuing authority, the date of issue, and any identifying information). Permits should encompass collection and, where applicable, export.*
Specimen deposition	*Indicate where the specimens have been deposited to permit free access by other researchers.*
Dating methods	*If new dates are provided, describe how they were obtained (e.g. collection, storage, sample pretreatment and measurement), where they were obtained (i.e. lab name), the calibration program and the protocol for quality assurance OR state that no new dates are provided.*

☐ Tick this box to confirm that the raw and calibrated dates are available in the paper or in Supplementary Information.

Ethics oversight	*Identify the organization(s) that approved or provided guidance on the study protocol, OR state that no ethical approval or guidance was required and explain why not.*

Note that full information on the approval of the study protocol must also be provided in the manuscript.

Animals and other research organisms

Policy information about studies involving animals; ARRIVE guidelines recommended for reporting animal research, and Sex and Gender in Research

Laboratory animals	Crosses between the Rosa26-CreER (CreER) C57BL/6 mice and conditional Ctcf allele (Ctcffl/fl) C57BL/6 mice were used for generation of BMDCs. Male and female 8-12 weeks old mice that were bred in specific pathogen-free facilities at Yonsei University College of Medicine were used for all experiments. with 12-hour light/dark cycles. Room temperature was maintained at 23±1°C and humidity level was controlled between 40-60%. Age- and sex-matched CreER littermate mice were used as wild-type (WT) controls throughout the study.
Wild animals	This study did not involve wild animals.
Reporting on sex	Both male and female mice were used since there was no difference in phenotype depending on sex and no sex-based analysis in our study.
Field-collected samples	This study did not involve field-collected samples.
Ethics oversight	All animal studied were approved by the Department of Laboratory Animal Resources Committee of Yonsei University College of Medicine.

Note that full information on the approval of the study protocol must also be provided in the manuscript.

Clinical data

Policy information about clinical studies

All manuscripts should comply with the ICMJEguidelines for publication of clinical research and a completedCONSORT checklist must be included with all submissions.

Clinical trial registration	*Provide the trial registration number from ClinicalTrials.gov or an equivalent agency.*
Study protocol	*Note where the full trial protocol can be accessed OR if not available, explain why.*
Data collection	*Describe the settings and locales of data collection, noting the time periods of recruitment and data collection.*
Outcomes	*Describe how you pre-defined primary and secondary outcome measures and how you assessed these measures.*

Dual use research of concern

Policy information about dual use research of concern

Hazards

Could the accidental, deliberate or reckless misuse of agents or technologies generated in the work, or the application of information presented in the manuscript, pose a threat to:

No	Yes	
☐	☐	Public health
☐	☐	National security
☐	☐	Crops and/or livestock
☐	☐	Ecosystems
☐	☐	Any other significant area

Experiments of concern

Does the work involve any of these experiments of concern:

No	Yes	
☐	☐	Demonstrate how to render a vaccine ineffective
☐	☐	Confer resistance to therapeutically useful antibiotics or antiviral agents
☐	☐	Enhance the virulence of a pathogen or render a nonpathogen virulent
☐	☐	Increase transmissibility of a pathogen
☐	☐	Alter the host range of a pathogen
☐	☐	Enable evasion of diagnostic/detection modalities
☐	☐	Enable the weaponization of a biological agent or toxin
☐	☐	Any other potentially harmful combination of experiments and agents

ChIP-seq

Data deposition

☒ Confirm that both raw and final processed data have been deposited in a public database such as GEO.

☒ Confirm that you have deposited or provided access to graph files (e.g. BED files) for the called peaks.

Data access links
May remain private before publication.

https://www.ncbi.nlm.nih.gov/geo/query/acc.cgi?acc=GSE185884
token: wzgpgmuclzgrfwz

Files in database submission

WT_rep1_1.fastq.gz
WT_rep1_2.fastq.gz
WT_rep2_1.fastq.gz
WT_rep2_2.fastq.gz
WT_rep3_1.fastq.gz
WT_rep3_2.fastq.gz
WTLPS_rep1_1.fastq.gz
WTLPS_rep1_2.fastq.gz
WTLPS_rep2_1.fastq.gz
WTLPS_rep2_2.fastq.gz
WTLPS_rep3_1.fastq.gz
WTLPS_rep3_2.fastq.gz
KO_rep1_1.fastq.gz
KO_rep1_2.fastq.gz
KO_rep2_1.fastq.gz
KO_rep2_2.fastq.gz
KO_rep3_1.fastq.gz
KO_rep3_2.fastq.gz
KOLPS_rep1_1.fastq.gz
KOLPS_rep1_2.fastq.gz
KOLPS_rep2_1.fastq.gz
KOLPS_rep2_2.fastq.gz
KOLPS_rep3_1.fastq.gz
KOLPS_rep3_2.fastq.gz

KOLPS_rep1_mm10_gencode.genes.results
KOLPS_rep2_mm10_gencode.genes.results
KOLPS_rep3_mm10_gencode.genes.results
KO_rep1_mm10_gencode.genes.results
KO_rep2_mm10_gencode.genes.results
KO_rep3_mm10_gencode.genes.results
WTLPS_rep1_mm10_gencode.genes.results
WTLPS_rep2_mm10_gencode.genes.results
WTLPS_rep3_mm10_gencode.genes.results
WT_rep1_mm10_gencode.genes.results
WT_rep2_mm10_gencode.genes.results
WT_rep3_mm10_gencode.genes.results
KOLPS_rnaseq_rep1.bw
KOLPS_rnaseq_rep2.bw
KOLPS_rnaseq_rep3.bw
KO_rnaseq_rep1.bw
KO_rnaseq_rep2.bw
KO_rnaseq_rep3.bw
WTLPS_rnaseq_rep1.bw
WTLPS_rnaseq_rep2.bw
WTLPS_rnaseq_rep3.bw
WT_rnaseq_rep1.bw
WT_rnaseq_rep2.bw
WT_rnaseq_rep3.bw
KO_CTCF_1.fastq.gz
KO_CTCF_2.fastq.gz
KO_H3K27ac_1.fastq.gz
KO_H3K27ac_2.fastq.gz
KO_H3K27me3_1.fastq.gz
KO_H3K27me3_2.fastq.gz
KO_H3K4me1_1.fastq.gz
KO_H3K4me1_2.fastq.gz
KO_H3K4me3_1.fastq.gz
KO_H3K4me3_2.fastq.gz
KO_input_1.fastq.gz
KO_input_2.fastq.gz
KOLPS_CTCF_1.fastq.gz
KOLPS_CTCF_2.fastq.gz
KOLPS_H3K27ac_1.fastq.gz
KOLPS_H3K27ac_2.fastq.gz
KOLPS_H3K27me3_1.fastq.gz
KOLPS_H3K27me3_2.fastq.gz
KOLPS_H3K4me1_1.fastq.gz
KOLPS_H3K4me1_2.fastq.gz
KOLPS_H3K4me3_1.fastq.gz
KOLPS_H3K4me3_2.fastq.gz
KOLPS_input_1.fastq.gz
KOLPS_input_2.fastq.gz
KOLPS_p65_1.fastq.gz
KOLPS_p65_2.fastq.gz
KOLPS_SMC1_1.fastq.gz
KOLPS_SMC1_2.fastq.gz
KO_p65_1.fastq.gz
KO_p65_2.fastq.gz
KO_SMC1_1.fastq.gz
KO_SMC1_2.fastq.gz
KO_STAT5_1.fastq.gz
KO_STAT5_2.fastq.gz
WT_CTCF_1.fastq.gz
WT_CTCF_2.fastq.gz
WT_H3K27ac_1.fastq.gz
WT_H3K27ac_2.fastq.gz
WT_H3K27me3_1.fastq.gz
WT_H3K27me3_2.fastq.gz
WT_H3K4me1_1.fastq.gz
WT_H3K4me1_2.fastq.gz
WT_H3K4me3_1.fastq.gz
WT_H3K4me3_2.fastq.gz

WT_input_1.fastq.gz
WT_input_2.fastq.gz
WTLPS_CTCF_1.fastq.gz
WTLPS_CTCF_2.fastq.gz
WTLPS_H3K27ac_1.fastq.gz
WTLPS_H3K27ac_2.fastq.gz
WTLPS_H3K27me3_1.fastq.gz
WTLPS_H3K27me3_2.fastq.gz
WTLPS_H3K4me1_1.fastq.gz
WTLPS_H3K4me1_2.fastq.gz
WTLPS_H3K4me3_1.fastq.gz
WTLPS_H3K4me3_2.fastq.gz
WTLPS_input_1.fastq.gz
WTLPS_input_2.fastq.gz
WTLPS_p65_1.fastq.gz
WTLPS_p65_2.fastq.gz
WTLPS_SMC1_1.fastq.gz
WTLPS_SMC1_2.fastq.gz
WT_p65_1.fastq.gz
WT_p65_2.fastq.gz
WT_SMC1_1.fastq.gz
WT_SMC1_2.fastq.gz
WT_STAT5_1.fastq.gz
WT_STAT5_2.fastq.gz
KO_CTCF_peaks_rmPATCH_p7.bed
KO_H3K27ac_peaks_rmPATCH.bed
KO_H3K27me3_peaks_rmPATCH.bed
KO_H3K4me1_peaks_rmPATCH.bed
KO_H3K4me3_peaks_rmPATCH.bed
KOLPS_CTCF_peaks_rmPATCH_p7.bed
KOLPS_H3K27ac_peaks_rmPATCH.bed
KOLPS_H3K27me3_peaks_rmPATCH.bed
KOLPS_H3K4me1_peaks_rmPATCH.bed
KOLPS_H3K4me3_peaks_rmPATCH.bed
KOLPS_p65_peaks_rmPATCH_rmblack.bed
KOLPS_SMC1_peaks_rmPATCH_p7.bed
KO_p65_peaks_rmPATCH_rmblack.bed
KO_SMC1_peaks_rmPATCH_p7.bed
WT_CTCF_peaks_rmPATCH_p7.bed
WT_H3K27ac_peaks_rmPATCH.bed
WT_H3K27me3_peaks_rmPATCH.bed
WT_H3K4me1_peaks_rmPATCH.bed
WT_H3K4me3_peaks_rmPATCH.bed
WTLPS_CTCF_peaks_rmPATCH_p7.bed
WTLPS_H3K27ac_peaks_rmPATCH.bed
WTLPS_H3K27me3_peaks_rmPATCH.bed
WTLPS_H3K4me1_peaks_rmPATCH.bed
WTLPS_H3K4me3_peaks_rmPATCH.bed
WTLPS_p65_peaks_rmPATCH_rmblack.bed
WTLPS_SMC1_peaks_rmPATCH_p7.bed
WT_p65_peaks_rmPATCH_rmblack.bed
WT_SMC1_peaks_rmPATCH_p7.bed
KO_CTCF.bw
KO_H3K27ac.bw
KO_H3K27me3.bw
KO_H3K4me1.bw
KO_H3K4me3.bw
KO_input.bw
KOLPS_CTCF.bw
KOLPS_H3K27ac.bw
KOLPS_H3K27me3.bw
KOLPS_H3K4me1.bw
KOLPS_H3K4me3.bw
KOLPS_input.bw
KOLPS_p65.bw
KOLPS_SMC1.bw
KO_p65.bw
KO_SMC1.bw

KO_STAT5.bw
WT_CTCF.bw
WT_H3K27ac.bw
WT_H3K27me3.bw
WT_H3K4me1.bw
WT_H3K4me3.bw
WT_input.bw
WTLPS_CTCF.bw
WTLPS_H3K27ac.bw
WTLPS_H3K27me3.bw
WTLPS_H3K4me1.bw
WTLPS_H3K4me3.bw
WTLPS_input.bw
WTLPS_p65.bw
WTLPS_SMC1.bw
WT_p65.bw
WT_SMC1.bw
WT_STAT5.bw
WT_HiC_rep1_R1.fastq.gz
WT_HiC_rep1_R2.fastq.gz
WT_HiC_rep2_R1.fastq.gz
WT_HiC_rep2_R2.fastq.gz
WTLPS_HiC_rep1_R1.fastq.gz
WTLPS_HiC_rep1_R2.fastq.gz
WTLPS_HiC_rep2_R1.fastq.gz
WTLPS_HiC_rep2_R2.fastq.gz
KO_HiC_rep1_R1.fastq.gz
KO_HiC_rep1_R2.fastq.gz
KO_HiC_rep2_R1.fastq.gz
KO_HiC_rep2_R2.fastq.gz
KOLPS_HiC_rep1_R1.fastq.gz
KOLPS_HiC_rep1_R2.fastq.gz
KOLPS_HiC_rep2_R1.fastq.gz
KOLPS_HiC_rep2_R2.fastq.gz
KO_HiC_rep1.allValidPairs.hic
KO_HiC_rep2.allValidPairs.hic
KOLPS_HiC_rep1.allValidPairs.hic
KOLPS_HiC_rep2.allValidPairs.hic
WT_HiC_rep1.allValidPairs.hic
WT_HiC_rep2.allValidPairs.hic
WTLPS_HiC_rep1.allValidPairs.hic
WTLPS_HiC_rep2.allValidPairs.hic
KO_K27Ac_HiChIP_rep1_R1.fastq.gz
KO_K27Ac_HiChIP_rep1_R2.fastq.gz
KO_K27Ac_HiChIP_rep2_R1.fastq.gz
KO_K27Ac_HiChIP_rep2_R2.fastq.gz
KOLPS_K27Ac_HiChIP_rep1_R1.fastq.gz
KOLPS_K27Ac_HiChIP_rep1_R2.fastq.gz
KOLPS_K27Ac_HiChIP_rep2_R1.fastq.gz
KOLPS_K27Ac_HiChIP_rep2_R2.fastq.gz
WT_K27Ac_HiChIP_rep1_R1.fastq.gz
WT_K27Ac_HiChIP_rep1_R2.fastq.gz
WT_K27Ac_HiChIP_rep2_R1.fastq.gz
WT_K27Ac_HiChIP_rep2_R2.fastq.gz
WTLPS_K27Ac_HiChIP_rep1_R1.fastq.gz
WTLPS_K27Ac_HiChIP_rep1_R2.fastq.gz
WTLPS_K27Ac_HiChIP_rep2_R1.fastq.gz
WTLPS_K27Ac_HiChIP_rep2_R2.fastq.gz
WTLPS_p65_HiChIP_rep1_R1.fastq.gz
WTLPS_p65_HiChIP_rep1_R2.fastq.gz
WTLPS_p65_HiChIP_rep2_R1.fastq.gz
WTLPS_p65_HiChIP_rep2_R2.fastq.gz
KO_K27Ac_HiChIP_rep1.allValidPairs.hic
KO_K27Ac_HiChIP_rep2.allValidPairs.hic
KOLPS_K27Ac_HiChIP_rep1.allValidPairs.hic
KOLPS_K27Ac_HiChIP_rep2.allValidPairs.hic
WT_K27Ac_HiChIP_rep1.allValidPairs.hic
WT_K27Ac_HiChIP_rep2.allValidPairs.hic

WTLPS_K27Ac_HiChIP_rep1.allValidPairs.hic
WTLPS_K27Ac_HiChIP_rep2.allValidPairs.hic
WTLPS_p65_HiChIP_rep1.hic
WTLPS_p65_HiChIP_rep2.hic
WTJSHLPS_RNA_rep1_1.fastq.gz
WTJSHLPS_RNA_rep1_2.fastq.gz
WTJSHLPS_RNA_rep2_1.fastq.gz
WTJSHLPS_RNA_rep2_2.fastq.gz
WTJSHLPS_RNA_rep3_1.fastq.gz
WTJSHLPS_RNA_rep3_2.fastq.gz
WTJSH_RNA_rep1_1.fastq.gz
WTJSH_RNA_rep1_2.fastq.gz
WTJSH_RNA_rep2_1.fastq.gz
WTJSH_RNA_rep2_2.fastq.gz
WTJSH_RNA_rep3_1.fastq.gz
WTJSH_RNA_rep3_2.fastq.gz
WTLPS_RNA_rep4_1.fastq.gz
WTLPS_RNA_rep4_2.fastq.gz
WTLPS_RNA_rep5_1.fastq.gz
WTLPS_RNA_rep5_2.fastq.gz
WTLPS_RNA_rep6_1.fastq.gz
WTLPS_RNA_rep6_2.fastq.gz
WT_RNA_rep4_1.fastq.gz
WT_RNA_rep4_2.fastq.gz
WT_RNA_rep5_1.fastq.gz
WT_RNA_rep5_2.fastq.gz
WT_RNA_rep6_1.fastq.gz
WT_RNA_rep6_2.fastq.gz
WTJSHLPS_RNA_rep1_mm10_gencode.genes.results
WTJSHLPS_RNA_rep2_mm10_gencode.genes.results
WTJSHLPS_RNA_rep3_mm10_gencode.genes.results
WTJSH_RNA_rep1_mm10_gencode.genes.results
WTJSH_RNA_rep2_mm10_gencode.genes.results
WTJSH_RNA_rep3_mm10_gencode.genes.results
WTLPS_RNA_rep4_mm10_gencode.genes.results
WTLPS_RNA_rep5_mm10_gencode.genes.results
WTLPS_RNA_rep6_mm10_gencode.genes.results
WT_RNA_rep4_mm10_gencode.genes.results
WT_RNA_rep5_mm10_gencode.genes.results
WT_RNA_rep6_mm10_gencode.genes.results
WTJSHLPS_RNA_rep1.bw
WTJSHLPS_RNA_rep2.bw
WTJSHLPS_RNA_rep3.bw
WTJSH_RNA_rep1.bw
WTJSH_RNA_rep2.bw
WTJSH_RNA_rep3.bw
WTLPS_RNA_rep4.bw
WTLPS_RNA_rep5.bw
WTLPS_RNA_rep6.bw
WT_RNA_rep4.bw
WT_RNA_rep5.bw
WT_RNA_rep6.bw
WT_H3K27ac_rep2_1.fastq.gz
WT_H3K27ac_rep2_2.fastq.gz
WT_H3K27ac_rep3_1.fastq.gz
WT_H3K27ac_rep3_2.fastq.gz
WTJSH_H3K27ac_rep1_1.fastq.gz
WTJSH_H3K27ac_rep1_2.fastq.gz
WTJSH_H3K27ac_rep2_1.fastq.gz
WTJSH_H3K27ac_rep2_2.fastq.gz
WTJSHLPS_H3K27ac_rep1_1.fastq.gz
WTJSHLPS_H3K27ac_rep1_2.fastq.gz
WTJSHLPS_H3K27ac_rep2_1.fastq.gz
WTJSHLPS_H3K27ac_rep2_2.fastq.gz
WTLPS_H3K27ac_rep2_1.fastq.gz
WTLPS_H3K27ac_rep2_2.fastq.gz
WTLPS_H3K27ac_rep3_1.fastq.gz
WTLPS_H3K27ac_rep3_2.fastq.gz

WT_H3K27ac_rep2.bw
WT_H3K27ac_rep3.bw
WTJSH_H3K27ac_rep1.bw
WTJSH_H3K27ac_rep2.bw
WTJSHLPS_H3K27ac_rep1.bw
WTJSHLPS_H3K27ac_rep2.bw
WTLPS_H3K27ac_rep2.bw
WTLPS_H3K27ac_rep3.bw
WTJSH_K27Ac_HiChIP_rep1_1.fastq.gz
WTJSH_K27Ac_HiChIP_rep1_2.fastq.gz
WTJSH_K27Ac_HiChIP_rep2_1.fastq.gz
WTJSH_K27Ac_HiChIP_rep2_2.fastq.gz
WTJSHLPS_K27Ac_HiChIP_rep1_1.fastq.gz
WTJSHLPS_K27Ac_HiChIP_rep1_2.fastq.gz
WTJSHLPS_K27Ac_HiChIP_rep2_1.fastq.gz
WTJSHLPS_K27Ac_HiChIP_rep2_2.fastq.gz
WT_K27Ac_HiChIP_rep3_1.fastq.gz
WT_K27Ac_HiChIP_rep3_2.fastq.gz
WT_K27Ac_HiChIP_rep4_1.fastq.gz
WT_K27Ac_HiChIP_rep4_2.fastq.gz
WTLPS_K27Ac_HiChIP_rep3_1.fastq.gz
WTLPS_K27Ac_HiChIP_rep3_2.fastq.gz
WTLPS_K27Ac_HiChIP_rep4_1.fastq.gz
WTLPS_K27Ac_HiChIP_rep4_2.fastq.gz
WTJSH_K27Ac_HiChIP_rep1.allValidPairs.hic
WTJSH_K27Ac_HiChIP_rep2.allValidPairs.hic
WTJSHLPS_K27Ac_HiChIP_rep1.allValidPairs.hic
WTJSHLPS_K27Ac_HiChIP_rep2.allValidPairs.hic
WT_K27Ac_HiChIP_rep3.allValidPairs.hic
WT_K27Ac_HiChIP_rep4.allValidPairs.hic
WTLPS_K27Ac_HiChIP_rep3.allValidPairs.hic
WTLPS_K27Ac_HiChIP_rep4.allValidPairs.hic

Genome browser session
(e.g. UCSC)

https://genome.ucsc.edu/s/kimhpemb/BMDC_mm10_submission

Methodology

Replicates

Two biological replicates were used for ChIP-seq dataset for each condition.

Sequencing depth

WT_input - total reads: 100758633, uniquely mapped reads: 100388943, read length: 101 bp, Paired end
WTLPS_input - total reads: 82126432, uniquely mapped reads: 81876647, read length: 101 bp, Paired end
KO_input - total reads: 78604961, uniquely mapped reads: 78127950, read length: 101 bp, Paired end
KOLPS_input - total reads: 74218957, uniquely mapped reads: 73765962, read length: 101 bp, Paired end
WT_CTCF - total reads: 103549782, uniquely mapped reads: 101704946, read length: 101 bp, Paired end
WTLPS_CTCF - total reads: 92806578, uniquely mapped reads: 91043018, read length: 101 bp, Paired end
KO_CTCF - total reads: 92257670, uniquely mapped reads: 90291082, read length: 101 bp, Paired end
KOLPS_CTCF - total reads: 87787256, uniquely mapped reads: 86092950, read length: 101 bp, Paired end
WT_H3K27ac - total reads: 29493768, uniquely mapped reads: 29409464, read length: 101 bp, Paired end
WTLPS_H3K27ac - total reads: 28697990, uniquely mapped reads: 28532532, read length: 101 bp, Paired end
KO_H3K27ac - total reads: 25619386, uniquely mapped reads: 25557786, read length: 101 bp, Paired end
KOLPS_H3K27ac - total reads: 28138504, uniquely mapped reads: 28066218, read length: 101 bp, Paired end
WT_H3K27me3 - total reads: 28320462, uniquely mapped reads: 28241668, read length: 101 bp, Paired end
WTLPS_H3K27me3 - total reads: 23094224, uniquely mapped reads: 23033388, read length: 101 bp, Paired end
KO_H3K27me3 - total reads: 27817818, uniquely mapped reads: 27718342, read length: 101 bp, Paired end
KOLPS_H3K27me3 - total reads: 24996420, uniquely mapped reads: 24847150, read length: 101 bp, Paired end
WT_H3K4me1 - total reads: 78985008, uniquely mapped reads: 76533496, read length: 101 bp, Paired end
WTLPS_H3K4me1 - total reads: 87311984, uniquely mapped reads: 84958654, read length: 101 bp, Paired end
KO_H3K4me1 - total reads: 86087662, uniquely mapped reads: 83862286, read length: 101 bp, Paired end
KOLPS_H3K4me1 - total reads: 64061830, uniquely mapped reads: 62345262, read length: 101 bp, Paired end
WT_H3K4me3 - total reads: 31729112, uniquely mapped reads: 31369184, read length: 101 bp, Paired end
WTLPS_H3K4me3 - total reads: 31176054, uniquely mapped reads: 30871756, read length: 101 bp, Paired end
KO_H3K4me3 - total reads: 31266322, uniquely mapped reads: 30894280, read length: 101 bp, Paired end
KOLPS_H3K4me3 - total reads: 30773396, uniquely mapped reads: 30382162, read length: 101 bp, Paired end
WT_p65 - total reads: 24111838, uniquely mapped reads: 23950898, read length: 101 bp, Paired end
WTLPS_p65 - total reads: 21041048, uniquely mapped reads: 20905932, read length: 101 bp, Paired end
KO_p65 - total reads: 21153326, uniquely mapped reads: 20894592, read length: 101 bp, Paired end
KOLPS_p65 - total reads: 23706676, uniquely mapped reads: 23493198, read length: 101 bp, Paired end
WT_SMC1 - total reads: 159816388, uniquely mapped reads: 157040814, read length: 101 bp, Paired end

	WTLPS_SMC1 - total reads: 153548672, uniquely mapped reads: 151362288, read length: 101 bp, Paired end<br>KO_SMC1 - total reads: 155761326, uniquely mapped reads: 153197042, read length: 101 bp, Paired end<br>KOLPS_SMC1 - total reads: 135094322, uniquely mapped reads: 132519888, read length: 101 bp, Paired end<br>WT_STAT5 - total reads: 37728018, uniquely mapped reads: 37271638, read length: 101 bp, Paired end<br>KO_STAT5 - total reads: 49208762, uniquely mapped reads: 48543916, read length: 101 bp, Paired end<br>WT_H3K27ac_rep2 – total reads: 108228258, uniquely mapped reads: 80002785, read length: 101 bp, Paired end<br>WT_H3K27ac_rep3 - total reads: 121417152, uniquely mapped reads: 89964588, read length: 101 bp, Paired end<br>WTJSH_H3K27ac_rep1 - total reads: 121024000, uniquely mapped reads: 91633907, read length: 101 bp, Paired end<br>WTJSH_H3K27ac_rep2 - total reads: 125628150, uniquely mapped reads: 94164526, read length: 101 bp, Paired end<br>WTJSHLPS_H3K27ac_rep1 - total reads: 125320934, uniquely mapped reads: 96502371, read length: 101 bp, Paired end<br>WTJSHLPS_H3K27ac_rep2 - total reads: 117850474, uniquely mapped reads: 89060114, read length: 101 bp, Paired end<br>WTLPS_H3K27ac_rep2 - total reads: 117959988, uniquely mapped reads: 87428502, read length: 101 bp, Paired end<br>WTLPS_H3K27ac_rep3 - total reads: 122013346, uniquely mapped reads: 87873118, read length: 101 bp, Paired end
Antibodies	anti-H3K27ac: Abcam ab4729, lot GR3251519-1<br>anti-H3K27me3: Abcam ab6002, lot GR3306698-2<br>anti-H3K4me1: Abcam ab8895, lot GR3264996-2<br>anti-H3K4me3: Abcam ab8580, lot GR288375-1<br>anti-SMC1: Bethyl lab A300-055A, lot 6<br>anti-CTCF: Cell Signaling Technology 2899, lot 2<br>anti-NF-κB p65: Cell Signaling Technology 8242, lot 13<br>anti-STAT5: Cell Signaling Technology 94205, lot 3
Peak calling parameters	peaks were called using the MACS2 tool callPeak, with the input provided for background correction, using the command -f BAMPE -g mm --nomodel to call peaks in histone modification or transcription factor. In case of H3K27ac and H3K27me3, we used the option --broad.
Data quality	Reads were filtered to remove PCR duplicates and reads which have lower MAPQ than 5 were removed. CTCF, SMC1 ChIP-seq peaks were filtered to threshold of FDR < 0.0000001 and H3K27ac, H3K27me3 ChIP-seq peaks were filtered to threshold of FDR < 0.00001 and rest of the peaks were used in default threshold of FDR.
Software	ChIP-seq data were aligned using BWA, converted SAM to BAM using samtools, removed duplicates using PICARD, and blacklist regions were removed using bedtools.

Flow Cytometry

Plots

Confirm that:

- [x] The axis labels state the marker and fluorochrome used (e.g. CD4-FITC).
- [x] The axis scales are clearly visible. Include numbers along axes only for bottom left plot of group (a 'group' is an analysis of identical markers).
- [x] All plots are contour plots with outliers or pseudocolor plots.
- [x] A numerical value for number of cells or percentage (with statistics) is provided.

Methodology

Sample preparation	Cell death and apoptosis were analyzed using an Annexin V/Propidium iodide (PI) staining kit (eBioscience). Cell proliferation was determined using CFSE staining. Aldehyde dehydrogenase (ALDH) activity was measured in the BMDCs using an ALDEFLUOR Kit (STEMCELL Technologies). For the intracellular detection of cytokine, cells were fixed and permeabilized with BD Cytofix/Cytoperm (BD Biosciences) and incubated with the relevant antibodies diluted in Perm/Wash buffer (BD Biosciences). Cytokines secreted into culture media were quantified using the CBA Mouse Inflammation Kit and the CBA Mouse Th1/Th2/Th17 Cytokine Kit (BD Bioscience). All samples were stained according to manufacturer's instructions.
Instrument	BD LSRFortessa, FACS VerseII (BD Bioscience) were used for data collection.
Software	Data acquisition: BD FACSuiteTM v1.0.6 and FACSDivaTM v8.0.2<br>Data analysis: FlowJo v10.5.3, BDTM Cytometric Bead Array FCAP Array Software v3.0
Cell population abundance	For MACS sorted cells, the purity of relevant population was validated by FACS analysis.
Gating strategy	Cells were gated for lymphocyte population (FSC-A/SSC-A), then singlets were gated in FSC-W/FSC-H and SSC-W/SSC-H and live cells were selected by Live/Dead Fixable Dye.<br>CD4+ T cells were assessed for expression of IFNγ and IL-17A. Dendritic cells were defined as CD11c+ and MHCII+. DC subsets were assessed for expression of CD11b, CD80, CD86, and for apoptosis, proliferation, ALDH activity.<br>For BM cell differentiation with GM-CSF, CD11c+MHCII+ cells were divided on the basis of CD11b and MHCII, which can further be sub-divided on the basis of CD135 and CD115. The gating strategy was illustrated in Supplementary Fig. 1a.

- [x] Tick this box to confirm that a figure exemplifying the gating strategy is provided in the Supplementary Information.

Magnetic resonance imaging

Experimental design

Design type	*Indicate task or resting state; event-related or block design.*
Design specifications	*Specify the number of blocks, trials or experimental units per session and/or subject, and specify the length of each trial or block (if trials are blocked) and interval between trials.*
Behavioral performance measures	*State number and/or type of variables recorded (e.g. correct button press, response time) and what statistics were used to establish that the subjects were performing the task as expected (e.g. mean, range, and/or standard deviation across subjects).*

Acquisition

Imaging type(s)	*Specify: functional, structural, diffusion, perfusion.*
Field strength	*Specify in Tesla*
Sequence & imaging parameters	*Specify the pulse sequence type (gradient echo, spin echo, etc.), imaging type (EPI, spiral, etc.), field of view, matrix size, slice thickness, orientation and TE/TR/flip angle.*
Area of acquisition	*State whether a whole brain scan was used OR define the area of acquisition, describing how the region was determined.*

Diffusion MRI ☐ Used ☐ Not used

Preprocessing

Preprocessing software	*Provide detail on software version and revision number and on specific parameters (model/functions, brain extraction, segmentation, smoothing kernel size, etc.).*
Normalization	*If data were normalized/standardized, describe the approach(es): specify linear or non-linear and define image types used for transformation OR indicate that data were not normalized and explain rationale for lack of normalization.*
Normalization template	*Describe the template used for normalization/transformation, specifying subject space or group standardized space (e.g. original Talairach, MNI305, ICBM152) OR indicate that the data were not normalized.*
Noise and artifact removal	*Describe your procedure(s) for artifact and structured noise removal, specifying motion parameters, tissue signals and physiological signals (heart rate, respiration).*
Volume censoring	*Define your software and/or method and criteria for volume censoring, and state the extent of such censoring.*

Statistical modeling & inference

Model type and settings	*Specify type (mass univariate, multivariate, RSA, predictive, etc.) and describe essential details of the model at the first and second levels (e.g. fixed, random or mixed effects; drift or auto-correlation).*
Effect(s) tested	*Define precise effect in terms of the task or stimulus conditions instead of psychological concepts and indicate whether ANOVA or factorial designs were used.*

Specify type of analysis: ☐ Whole brain ☐ ROI-based ☐ Both

Statistic type for inference (See Eklund et al. 2016)	*Specify voxel-wise or cluster-wise and report all relevant parameters for cluster-wise methods.*
Correction	*Describe the type of correction and how it is obtained for multiple comparisons (e.g. FWE, FDR, permutation or Monte Carlo).*

Models & analysis

n/a	Involved in the study
☐	☐ Functional and/or effective connectivity
☐	☐ Graph analysis
☐	☐ Multivariate modeling or predictive analysis

Functional and/or effective connectivity	*Report the measures of dependence used and the model details (e.g. Pearson correlation, partial correlation, mutual information).*
Graph analysis	*Report the dependent variable and connectivity measure, specifying weighted graph or binarized graph, subject- or group-level, and the global and/or node summaries used (e.g. clustering coefficient, efficiency, etc.).*

Multivariate modeling and predictive analysis

Specify independent variables, features extraction and dimension reduction, model, training and evaluation metrics.
