# Supplementary material for: CTCF controls three-dimensional enhancer network underlying the inflammatory response of bone marrow-derived dendritic cells
Source: Nat Commun. 2023 Mar 8;14:1277. doi: 10.1038/s41467-023-36948-5 (PMC9992691; doi:10.1038/s41467-023-36948-5)

**Figure 1c**

**CTCF**

250 kDa

130 kDa


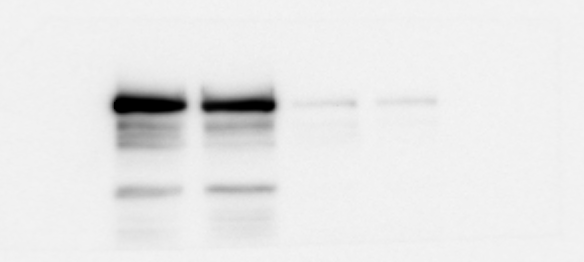


**β-actin**

35 kDa

55 kDa


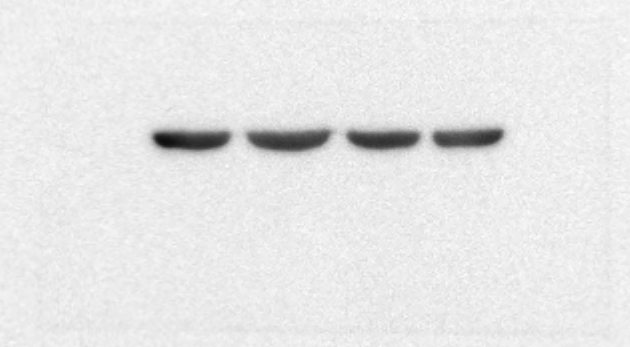


**Figure 4i**

**CTCF**

250 kDa

130 kDa


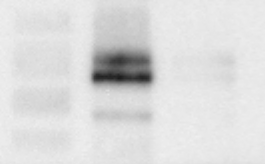


**ALDH1A2**

55 kDa

70 kDa


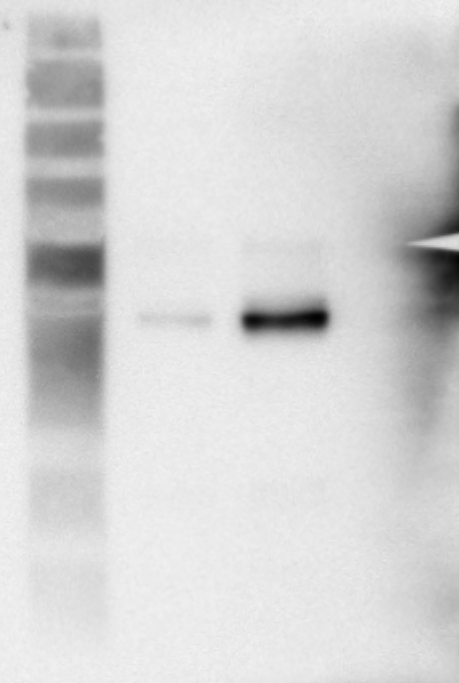


**β-actin**

55 kDa

35 kDa


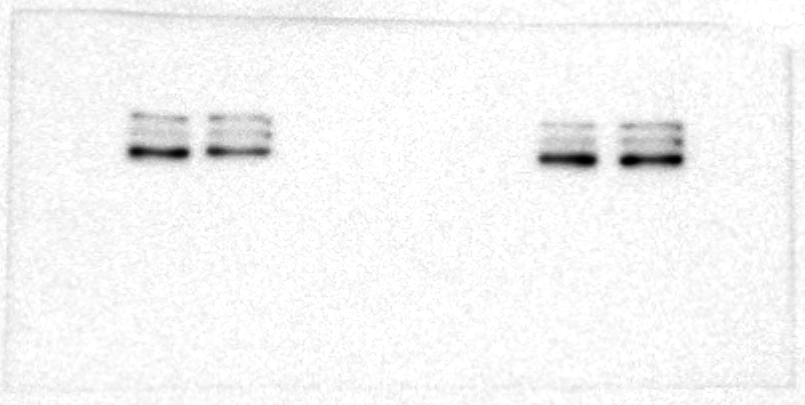


**Figure 5a**

250 kDa

**CTCF**

130 kDa


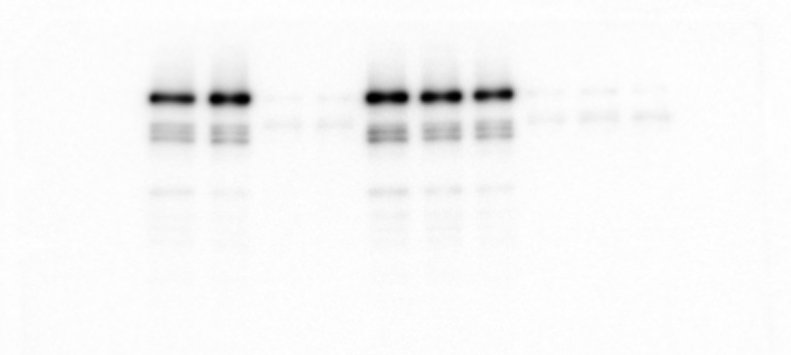


**p-JAK2**

100 kDa

130 kDa


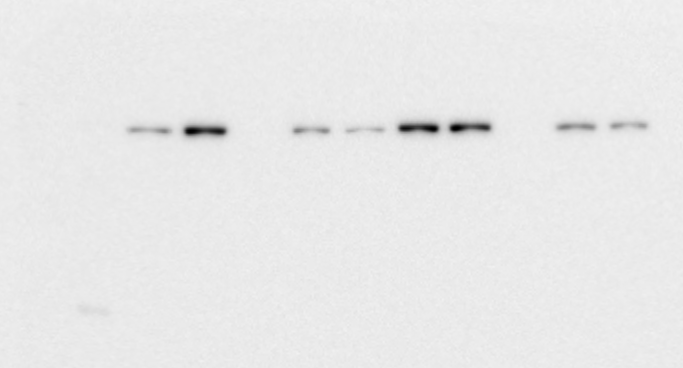


**JAK2**

100 kDa

130 kDa


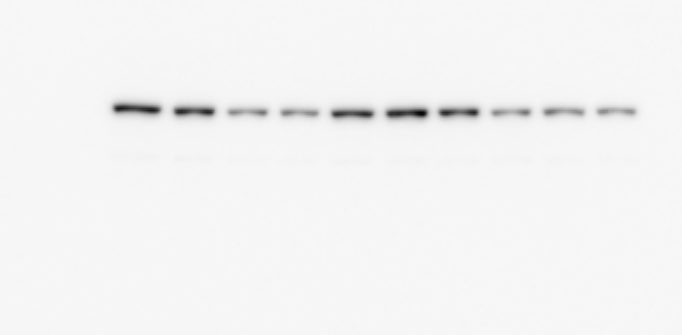


**p-STAT5**

70 kDa

100 kDa


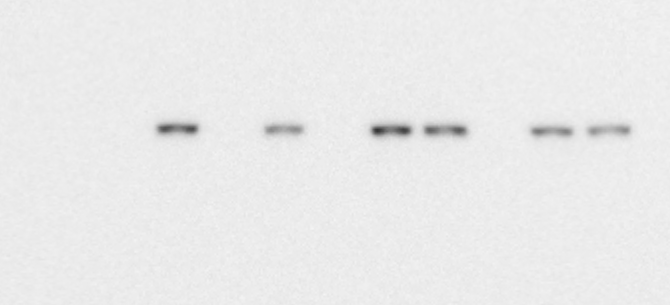


**STAT5**

70 kDa

100 kDa


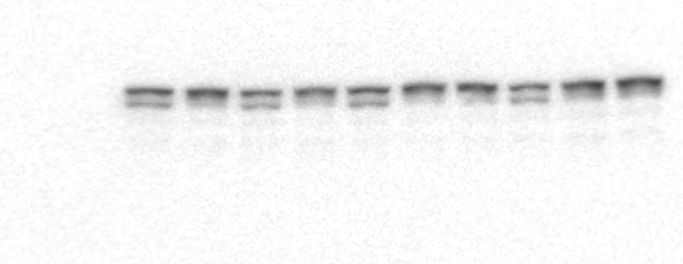


**β-actin**

55 kDa

35 kDa


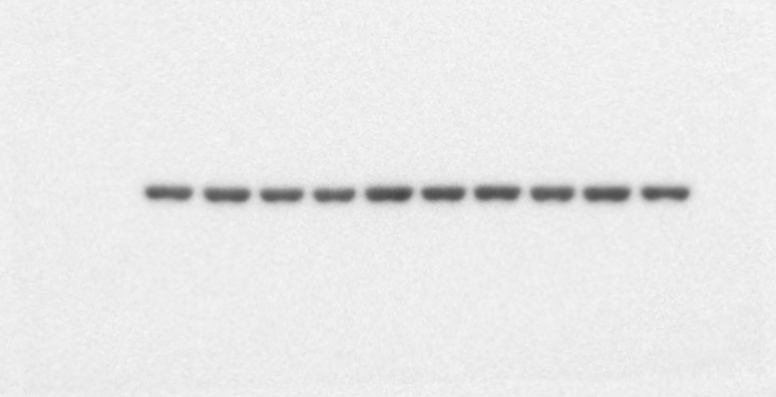


**Figure 6a**

**p-IKKα/β**

70 kDa


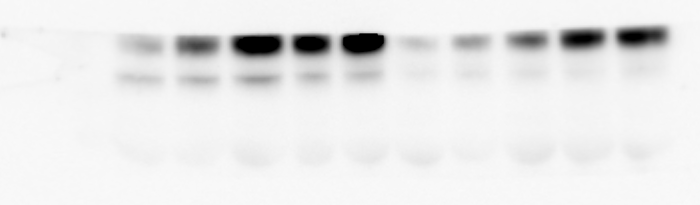


**IKKα**

55 kDa

100 kDa

70 kDa


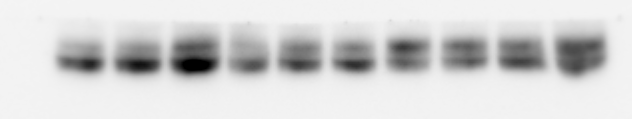


**p-IκBα**

55 kDa

35 kDa


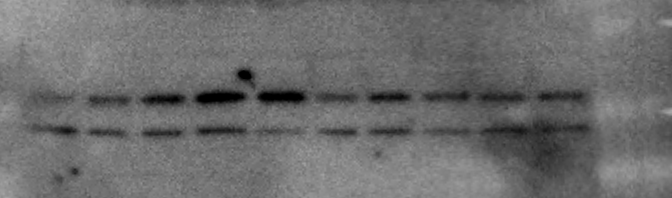


**IκBα**

35 kDa


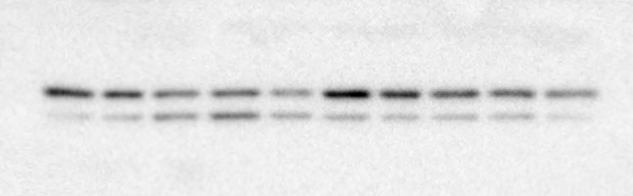


**RelA**

70 kDa


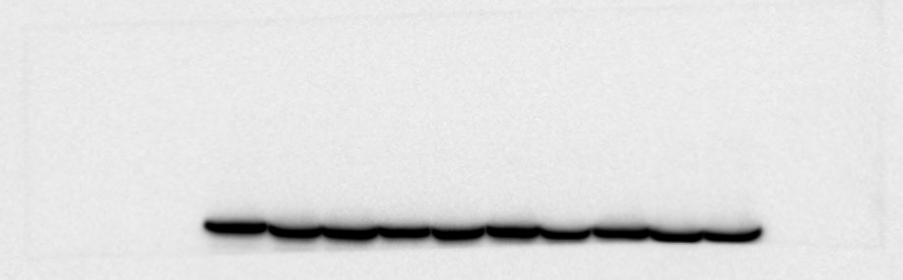


**β-actin**

250 kDa

35 kDa

55 kDa


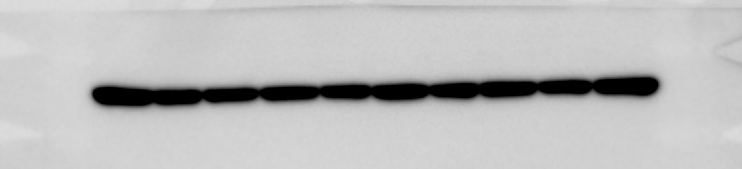


**
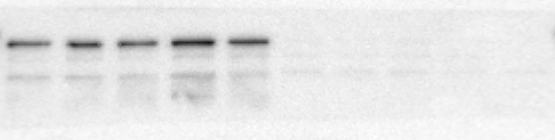
 CTCF**

130 kDa

**Figure 6d**

**RelA**

70 kDa

55 kDa


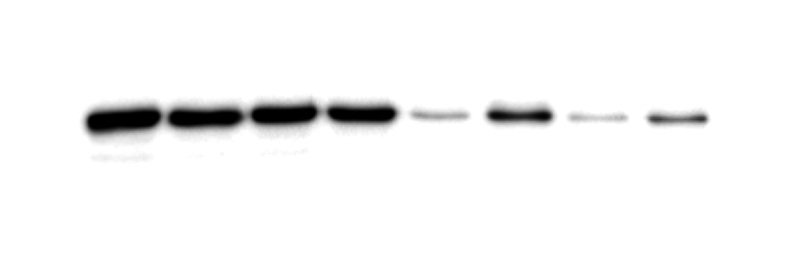


**Lamin B1**

70 kDa


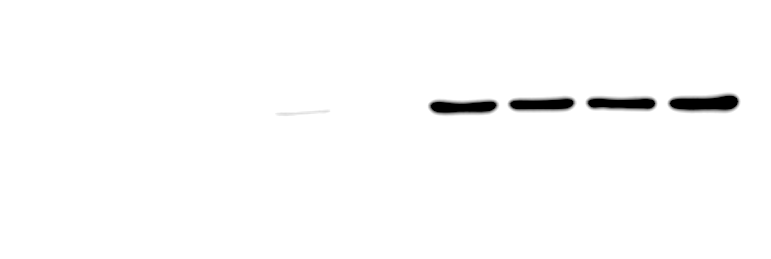


**α-tubulin**

55 kDa


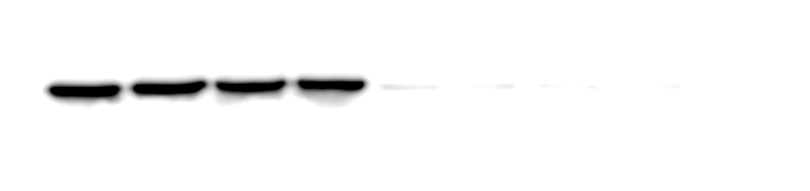


**Figure 8a**

**CTCF**

170 kDa

130 kDa


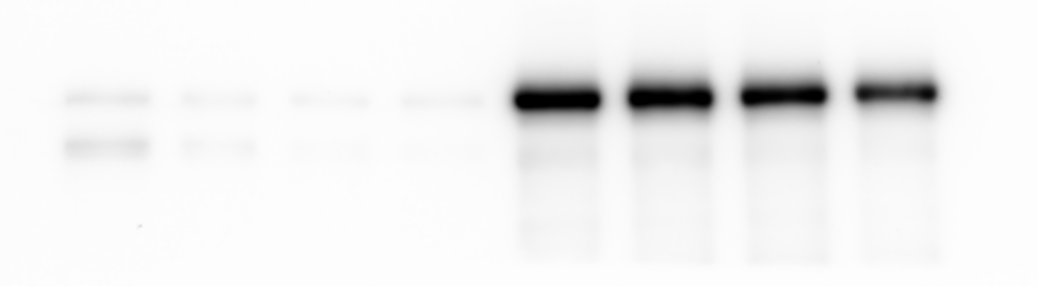


**RelA**

55 kDa

70 kDa


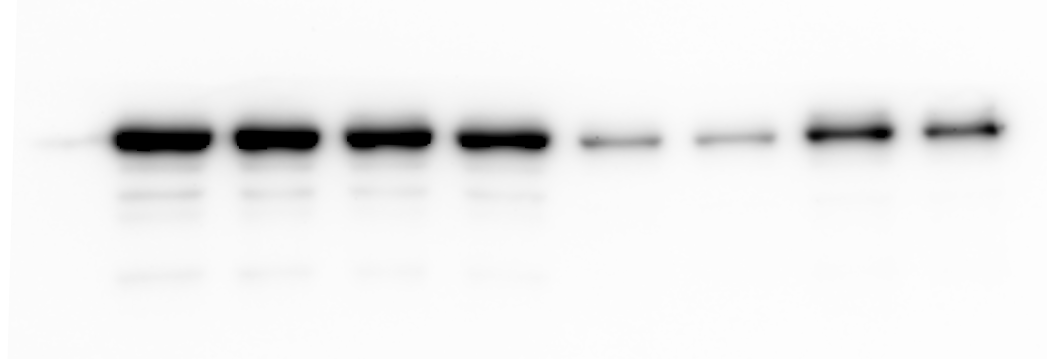


**LaminB1**

70 kDa


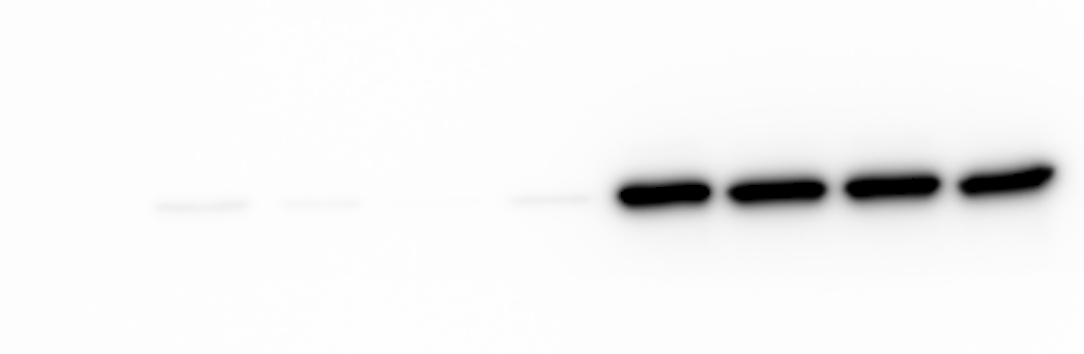


**α-tubulin**

55 kDa


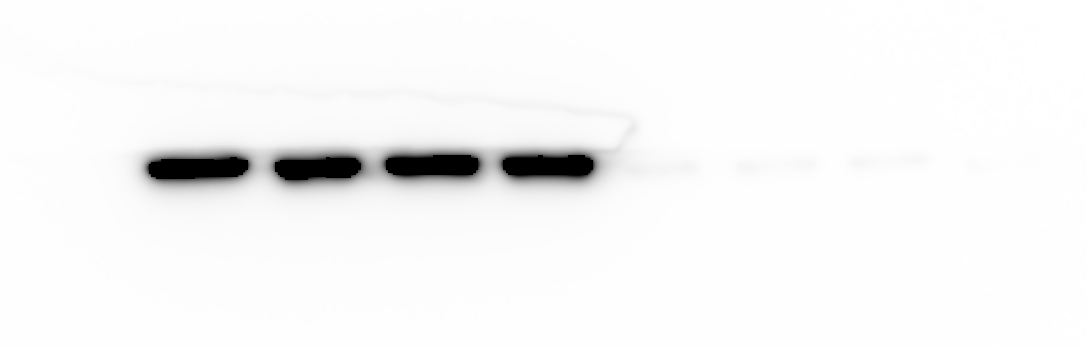


**Figure S7b**

**p-STAT5**

130 kDa

95 kDa


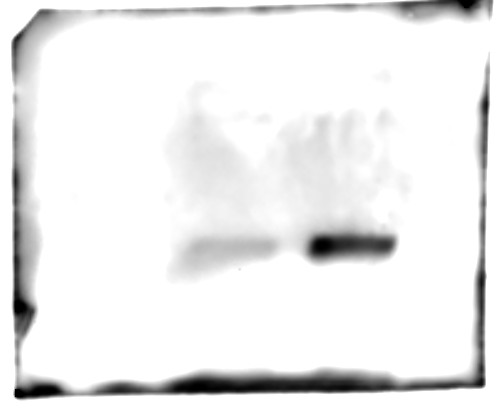


**STAT5**

130 kDa

95 kDa


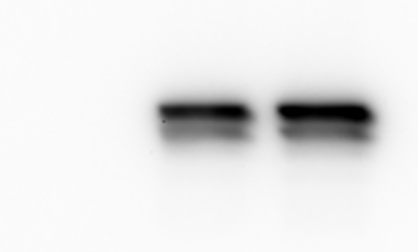


**β-actin**

56 kDa

43 kDa


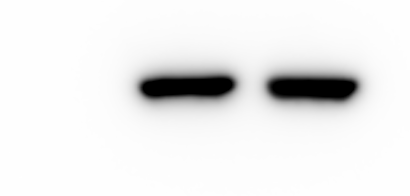

Supplement: Supplementary file 4 — Source Data [file 41467_2023_36948_MOESM4_ESM.zip › DC-CTCF_Nature_Comm_Revision_2nd_Source data_unprocessed images.docx]
